# VOCs and Odor Episodes near the German–Czech Border: Social Participation, Chemical Analyses and Health Risk Assessment

**DOI:** 10.3390/ijerph19031296

**Published:** 2022-01-24

**Authors:** Jan Leníček, Ivan Beneš, Eva Rychlíková, David Šubrt, Ondřej Řezníček, Tomáš Roubal, Joseph P. Pinto

**Affiliations:** 1 Health Institute (Zdravotní Ústav), 40001 Ústí nad Labem, Czech Republic; eva.rychlikova@gmail.com (E.R.); david.subrt1@gmail.com (D.Š.); ondrej.reznicek@zuusti.cz (O.Ř.); tomas.roubal@zuusti.cz (T.R.); 2 Department of Environmental Science and Engineering, University of North Carolina, Chapel Hill, NC 27599, USA; joepinto@ad.unc.edu

**Keywords:** odorous compounds, canister sampling, passive sampling, GC-MS analysis, health impact

## Abstract

People living on both sides of the German–Czech border are subject to episodes of odor air pollution. A joint German–Czech air sampling and risk assessment project was established to identify the substances responsible and their sources. Twenty-four volunteer study participants, 14 from the NW Czech Republic and 10 from Germany (Saxony) reported odors and collected canister samples during sampling periods in winter 2017 and 2018 and autumn 2018. Canister samples and passive samplers were analyzed for volatile organic compounds (VOCs) and passive samplers were analyzed for VOCs and carbonyls. OAVs (Odor Activity Values) and back trajectories were calculated with the aim of identifying the odor sources. Calculated OAVs were in excellent agreement with perceived smells close to an oil processing plant. Odorants identified in fifty canister samples during odor episodes and carbonyl measurements close to the edible oil processing plant were used for health evaluation. Odors reported by participants in Saxony frequently differed from those reported by participants in the Czech Republic. This suggests that certain sources of odor lying on either side of the border only affect that side and not the other with similar considerations regarding health effects. VOCs, including carbonyls, were also sampled at two relatively remote locations during winters of 2017 and 2018; two main sources of odorous compounds were identified at these sites. Analysis of samples taken at sampling sites shows that VOC air pollution and, to a lesser extent carbonyl pollution, originate from both industrial and local sources. Even though levels of sampled substances were not associated with acute effects at any site, long-term exposures to selected compounds could be cause for concern for carcinogenicity at some sites. Odors in Seiffen were associated with carcinogenic compounds in can samples. Although not necessarily representative of long-term exposures to the compounds studied, results such as these suggest that further study is needed to better quantify long-term exposure to potentially harmful compounds, and to either confirm or deny the existence of substantive health risk.

## 1. Introduction

Odorous compounds, including many volatile organic compounds (VOCs), play an important role in air pollution in industrial areas and the residential areas surrounding them. Odors may cause a variety of undesirable reactions in people. The monitoring of odorous compounds in ambient air is an important task for environmental research because malodorous compounds could also be toxic. Even if odor causing chemicals are not toxic, they can affect the quality of life. Exposure to noxious odors can generate reactions ranging from emotional stresses such as unease and discomfort to headaches, respiratory problems, nausea, or vomiting. Generally, the impact of an odor results from a combination of interacting factors—FIDOL, namely frequency (F), intensity (I), duration (D), offensiveness (O), and location (L). This involves the introduction of an objective odor limit: “Facilities that are identified as sources of offensive odors shall ensure that 10-minute average concentration of odor resulting from all sources at the facility and determined in accordance with accepted procedures, shall be less than 1 odor unit 99.5% of time at the most impacted sensitive receptor” [1]. Odors are regulated to different degrees in the European Union [2]. In Germany, all types of odors produced from industrial and animal processing, but not traffic, residential heating, manure spreading or vegetation, are considered to be annoying or unpleasant according to the Bundes Immissionsschutz Gesetz. In the Czech Republic, on the other hand, there are no guidelines or regulations used at the federal level and discretion is given to local authorities in dealing with complaints of malodors.

A large number of studies have been conducted examining the relationships between noxious odors and concentrations of pollutants emitted from sources, such as manufacturing facilities [3], motor vehicles, in particular diesels [4,5], petrochemical plants [6], industrial facilities, landfills [7,8,9,10,11], water treatment plants, waste combustion, and animal processing [12,13,14,15].

The aim of the current study is to identify compounds and their sources associated with noxious odors, especially those that could pose health risks to the local population. In this study, VOCs, which include hydrocarbons, aldehydes, ketones, esters, and halogenated organic compounds were measured at several sites near the border between the Northwestern Czech Republic and Saxony (Germany). Note that the emissions of many of these compounds are regulated as hazardous air pollutants (HAPS) by the U.S. Environmental Protection Agency (EPA); they are designated as toxic air pollutants that are known or suspected to cause cancer or other serious health effects, such as reproductive effects or birth defects, or adverse environmental effects [16].

The study area was chosen from a historical perspective. Prior to the early 1990s, the German–Czech border region was characterized by very poor air quality due to intensive industrial and domestic brown coal combustion. Even though air quality has steadily improved since then, the region is still subjected to episodes of air pollution [17], and complaints of inhabitants about odorous compounds are still registered with local authorities in the region.

Odor episodes in the area occur on individual days, especially in winter, and often in conjunction with stagnant weather conditions. Despite various investigations over the years, it has not yet been possible to attribute the occurrence of odors to specific sources. In addition to industrial facilities in the Czech Basin, power plants supplying domestic heating in the region are also a source of pollution. According to the perception of the citizens concerned and the reports of the authorities, it is not just one, but several different odors that are typically involved.

There are many industrial areas of the Czech Republic in the Ore Mountains region. Open pit brown coal mines in the vicinity of Bílina, Most and Sokolov, are sources of fuel for the Ledvice, Počerady, Most, and Vřesová lignite-fired power plants. There are petrochemical industry facilities in Záluží by Litvínov, chemical industry facilities in Ústí nad Labem and Sokolov, and all told, there are about 100 industrial facilities in this region.

On the Saxon side of the Ore Mountains, the situation is significantly more diverse. A large number of smaller companies can be found here and can be a source of odors. There are a total of 46 manufacturing facilities in eleven municipalities in the vicinity of the border. These include farms, livestock, gas and wood processing plants, paint shops, recycling plants, and plants that burn and process oil, coal and fuel oil, and plastics. Heating facilities of apartment buildings on both sides of the border, which use solid fuels such as wood and coal, can contribute to odor problems.

Czech scientists collected VOC samples in canisters at several locations and on passive samplers during three sampling periods, 01–03/2017, 11/2017–03/2018 and 11–12/2018 at two rural, background monitoring sites, Lom u Mostu (LOM) (Czech Republic) and Deutschneudorf (DND) (Germany). Sampling at another background site, Jeřabina (JER) (on the German–Czech border), was added for passive sampling of carbonyls in the final period, 15.11.2017–27.02.2018. These background sites were chosen because the area has historically been characterized by poor air quality. Measurement of carbonyls in Ústí nad Labem close to the edible processing plant was added in the period 27 September–18 October 2018 as many complaints on odorous compounds were registered in this town by local authorities. In addition, odors were also categorized to help determine compounds, which could also pose health risks. Special attention is paid to compounds emitted by vehicular traffic, solid fuel combustion, and industrial and agricultural sources in addition to transboundary transport. Sampling methodology for both chemical measurements and odor records from volunteer participants in the affected area are described in Section 2. Results of air pollution measurements and odor characterizations by the volunteer participants are given in Section 3. A health risk assessment due to exposure to carcinogenic and otherwise harmful compounds is given in Section 4.

## 2. Materials and Methods

### 2.1. Selection of Volunteer Participants and Their Role in the Project

Twenty-four volunteers living in the vicinity of the German–Czech border, fourteen from the Czech Republic and ten from Germany were selected for this project to report malodorous episodes. Demographic characteristics (age, sex) for the participants are given in Table S1 in the Supplementary Materials. All participants were tested for their physiological state including the sense of smell using dynamic olfactometry. The study participants recorded odor during 3 periods (01–03/2017, 11/2017–03/2018, 11–12/2018). The odor records comprised date and time of odor perception, locality, odor characteristics and intensity (3-level scale), and subjective physical symptoms during odor perception until the end of that day. If the study participants could positively determine the odor source, they described it. Participants could choose from several predetermined characteristic odors or they could describe the odor in their own words (item “other character” and a description). This question had multiple responses. Similarly, there were multiple replies to the question about physical symptoms (including the item “without symptom”), but the respondent could describe his symptom in his own words (item “other symptom” and a description). Individual participants varied in the intensity of their active participation. Some recorded odors only during one period, while others during two or three periods. Each day, participants noted when they were not actively monitoring (e.g., when they left the study area or when they temporarily lost the sense of smell due to illness).

The average age of the ten volunteers in Germany was 61 and it was 44 for the fourteen volunteers in Czechia. The health status of volunteers was not monitored because they were selected based on the results of dynamic olfactometry showing they had comparable sensitivity to odors.

Fourteen of the participants (five from the Czech Republic and nine from Germany) were equipped with evacuated Silco-Can canisters for sampling VOCs when such odors were noted. Samples were transported to the laboratory, where they were transferred into adsorbent tubes, which were then thermally desorbed and analyzed by GC-MS. OAV values and back trajectories were calculated with the aim of identifying the odor sources.

### 2.2. Monitoring Sites in the Study Area

**Figure 1:**
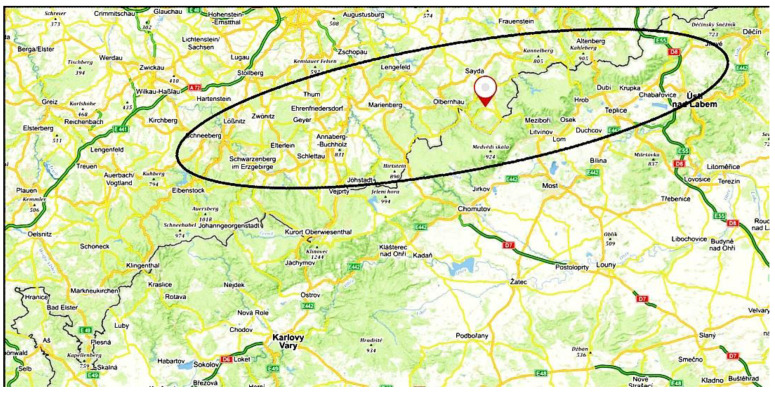
Map of the Northwestern quadrant of the Czech Republic and Southern Saxony. The oval indicates the approximate study area where data was collected. The town of Seiffen in Saxony is denoted by the red balloon. (Background map: mapy.cz).

A map showing the general location of the study area is given in Figure 1.

The terrain is generally mountainous with the Ore Mountain Range (Erzgebirge, Krušné hory) lying along the main axis of the ellipse shown in Figure 1. Locations and descriptions of fixed air quality monitoring sites in the study area are given in Table 1. In addition to the long-term, stationary monitoring measurements, passive sampling of VOCs was added at the two rural background sites (Deutschneudorf (DE) and Lom u Mostu (LOM) for all three sampling periods; sampling was also carried out at Jeřabina (JER) (which did not have routine monitoring capability because of a lack of power) during the third sampling period.

### 2.3. Sampling and Analysis of VOCs

Fifty Silco Can canisters with volume 6 L and 3 L (Restek) were cleaned and evacuated. Volunteer participants involved in the project were trained in the use of the evacuated Silco Can canisters for collecting VOC samples. As soon as odor was registered by one of the volunteer participants, a sample was collected over several minutes, until the container reached equilibrium with atmospheric pressure. Generally, this approach is used when unknown analytes must be identified, when the air contains high concentrations of analytes at certain (short) times, or when an odor is noticed, and a sample must be obtained quickly. Samples were transported to the laboratory at the Zdravotní Ústav in Ústí nad Labem as soon as possible, generally within 1–3 days. Detailed laboratory procedures for extracting and analyzing canister samples as well as procedures for carbonyl compounds are described in the Supplementary Materials. Standards used for analyzing hydrocarbons are shown in Table S2.

Radiello^TM^ 120 diffusive air samplers and Radiello^TM^ 145 BTEX/VOC cartridges for thermal desorption were used for passive sampling of VOCs other than carbonyls. Sampling rate values at 298 K (Q_298_) and 1013 hPa, used to derive sample volumes, were based on experimentally measured values for 77 compounds in a standard atmosphere chamber, which were given by the supplier [24]. The diffusion coefficients for other compounds were calculated using the EPA calculator [25]; the sampling rate was calculated according to Equation (1):

(1) Q=Kd×60D

where Q is the sampling rate mL·min^−1^, D is the diffusion coefficient cm^2^·s^−1^, and Kd is the experimentally determined effective length 14.145 ± 0.110 cm for the RAD 120 diffusive body.

The sampling rate, Q, is a function of the diffusion coefficient D, which is a thermodynamic property of each chemical substance. D varies with temperature (T) and pressure (p); therefore, the sampling rate is also a function of those variables.

Sampling rates vary from the value at 298 K and the effect of temperature is expressed by Equation (2):

Q_T_ = Q_298_(T/298)^1.5^ (2)

where Q_K_ is the sampling rate at temperature T and Q_298_ is the reference value at 298 K.

The correction of Q for atmospheric pressure is usually negligible [24]. Cartridges were exposed for 8 days and the mean temperature over the sampling period was calculated. The analyses of the sample cartridges used a thermal desorption system (TD Unity Markes) coupled to gas chromatograph (HP 6890 Agilent). Thermal desorption of VOC was performed in several steps: The sampling tube was desorbed at 300 °C and released VOCs were flushed to a trap. Further details of the laboratory procedures for extracting and analyzing passive samples including standards used for analyzing hydrocarbons and procedures for carbonyl compounds are described in the Supplementary Materials.

Radiello^®^ 1201 diffusive air samplers and 165 Radiello^®^ cartridge adsorbents with 2,4-dinitrophenylhydrazine (DNPH) coated FLORISIL^®^ were used for passive sampling of carbonyl compounds. The carbonyls were trapped, making them react with DNPH to form the corresponding 2,4-dinitrophenyl hydrazone derivatives. Sampling rate values at 298 K (Q_298_) and 1013 hPa, for formaldehyde, acetaldehyde, acrolein, propionaldehyde, butanal, isopentanal, pentanal, and hexanal are given by the supplier [26]. For other carbonyls, diffusion coefficients were calculated and the sampling rates were estimated using Equation (3):

(3) $$Q_{U}=Q_{K}\times D_{U}/D_{K}$$

where Q_U_ is unknown sampling rate for analyte U, Q_K_ is known sampling rate for the analyte K, and D_U_ and D_K_ are diffusion coefficients for analytes U and K.

The Q_hexanal_ value was used for the aliphatic carbonyls (heptanal, octanal, nonanal, and decanal) calculation; benzaldehyde was used for aromatic carbonyls (o-tolualdehyde, m-tolualdehyde, p-tolualdehyde, and dimethyl benzaldehyde); acrolein was used for unsaturated carbonyls (methacrolein and crotonaldehyde), and butanone was used for acetone. The sampled material was eluted from the cartridges by washing it with 2 mL acetonitrile and diluted with 2 mL of ultrapure water. Detection was by HPLC-UV/VIS detector at 365 nm. More detailed information about the laboratory procedures for extracting and analyzing passive samples, including standards used for analyzing carbonyl compounds, are given in the Supplementary Materials.

#### Measurement of Odors

Environmental odors were quantified by chemical measurements coupled with information for their odor thresholds. This method is more readily carried out than olfactometric analysis, and so was used in this study for odor analysis. The method is based on trapping compounds in a cartridge packed with sorbent. It is well developed for volatile organic compounds and is applicable for sampling of odorous VOC compounds with subsequent gas chromatography/mass spectrometry analysis [3,27,28,29,30,31].

In order to obtain information about odors based on the results of chemical analyses, the Odor Activity Value (OAV) must be calculated. The OAV represents the sum of the concentrations of potentially odorous compounds weighted by their odor threshold (OT) [11,32], values for which are taken from the literature. Equation (4) was used for the calculation of OAV:

(4) $$\mathrm{OAV}=\overset{n}{\underset{i=1}{\sum }}\mathrm{Ci}/\mathrm{OTi}$$

where OAV = Odor Activity Value (ou), Ci = Concentration of compound i (ppb),OT = Odor Threshold of compound i (ppb·ou^−1^), and ou = odor unit.

It should be noted here that these two approaches, i.e., pollutant measurements with OTs and olfactometry, can give substantially different results with low correlation between values using these two techniques. The main problem in using chemical measurements to evaluate OAVs is that the odor threshold concentrations found in the literature often differ by several orders of magnitude [32]. The large differences among OT values in the literature are due to different methodologies used to obtain them, e.g., odor thresholds for some compounds can be several orders of magnitude lower when using a dynamic system as opposed to a static system [33]. In this study, odorant concentrations were converted to their OAV using the OT databases in which values were generated using dynamic dilution olfactometry [11,32,33,34,35].

### 2.4. Odor Data Analysis

**Figure 2:**
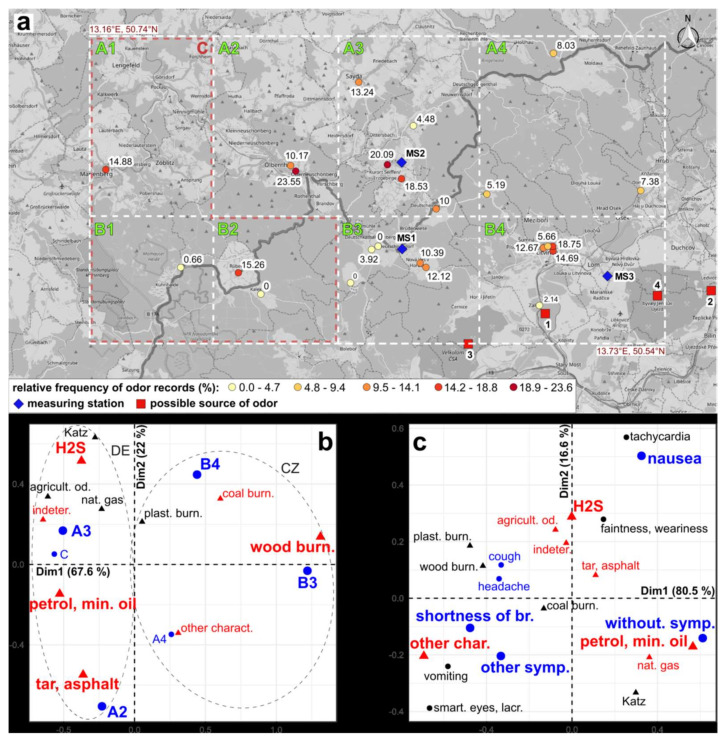
(**a**) Geographical distribution of study participants together with relative frequency of odor records (%). Measuring station: MS1–Deutschneudorf, MS2–Schwartenberg, MS3–Lom. Possible source of odor: 1–Unipetrol, 2–coal-fired power station Ledvice, 3—open pit mine ČSA, 4—open pit mine Bílina. There are drawn spatial segments (A1, …, A4, B1, …, B4, C = A1 + B1 + B2) of investigated area in the map, too. (Source of background map: https://openstreetmap.cz, accessed on 5 December 2021 14:10); (**b**) CA biplots: association between odor characteristics and spatial segments of study area; (**c**) CA biplots: association between odor characteristics and physical symptoms. Active elements are displayed in color; supplementary elements are displayed in black.

The percentage of days with odor records over the total number of observation days (relative frequency of odor records) for each participant was determined, and the permutation-based t-test was used to compare the frequency between Czech and German participants. To analyze association between geographical location and odor characteristics, the investigated area was divided into eight spatial segments (A1 to A4, B1 to B4 in Figure 2). Subsequently, the association of odor characteristics with the eight segments was explored using the chi-squared test and the correspondence analysis (using the “CA“ function from the R package, FactoMineR [36]. Correspondence analysis (CA) is an ordination method that examines the interrelationships of the categories of two qualitative variables. One of the outputs is a biplot, which graphically illustrates these relationships to the ordination plane. The closer the categories are, the more they are associated with each other. The further away the categories are from the intersection of the ordination axes, the more they distort the model of independence of both variables, i.e., they are those categories that are associated with each other and not with others. In order to eliminate distortion, low frequency categories were considered as supplementary elements. This means that they do not affect the position of the ordination axes as they are displayed in the biplot based on the position of the active elements [37,38]. We applied the same approach to explore the association between odor characteristic and physical symptoms of the participants. Statistical computations were performed in R v. 3.5.1 [39].

## 3. Results of the Sampling Program

### 3.1. Odor Monitoring by Volunteer Participants

Participants recorded a total of 491 observations of odor air pollution. A total relative frequency of odor records was 9.4% for all Czech participants and 13.3% for all German participants.

The highest relative frequencies of odor reports were recorded in Olbernhau, Seiffen (DE), and Litvínov (CZ). At some locations, we also recorded zero values (Hora Svaté Kateřiny, Kalek, etc.) The relative frequencies of odor records made by each participant are shown in Figure 2a. The frequency of odor reports by German participants might appear to be higher than in the Czech Republic, however, the difference was not statistically significant (CZ: 11.8 ± 10.64%, DE: 12.9 ± 7.14%).

The odor characteristics are listed in Table 2.

The most frequently encountered odor descriptor was “petrol, mineral oil” followed by “hydrogen sulfide” in Table 2, part (a). However, H_2_S was not measured in the present study. There were notable differences in odors perceived by participants in either Czechia or Germany. Most reports by participants in Czechia were of coal, wood, and plastic burning. On the other hand, most reports by participants in Germany were of petrol, mineral oil, tar and asphalt, natural gas and Katzendreck (cat feces). (Katzendreck is a term used mainly on the Saxon side of the Ore Mountains and originally may have included mostly malodprous sulphur substances from coal burning such as mercaptans. It was later adopted to describe many odors of different origins. It is in widespread use today, without being precisely defined. Participants in Germany responded overwhelmingly to indeterminate odors and to those characterized as agricultural. The category “other odor descriptors” (29.5% of records) mainly include: cowshed (12.2%); chemical odor (12.2%); soot and smoke (9.1%); soot/chemical odor (7%); burnt gum (5%); slurry (4%); oil odor (3%); incineration of construction waste (2%). Associations with odors include: south-easterly wind (11.2%) and temperature inversion (3%). Physical symptoms associated with various odors were reported by a little less than half of participants. The major categories were headache, cough, and shortness of breath. Apart from the more distinguishable symptoms given in Table 2, part (b), other symptoms were actually the major category.

Exploring the association between odor descriptors and spatial segments, we faced the problem of low theoretical frequencies in segments A1, B1, B2 (Figure 2a). Therefore, we unified these segments into one (segment C). The same problem occurred for descriptors whose percentage was below 10% in Table 2, part (a) (i.e., “plastic burning”, “agricultural odor”, “natural gas”, and “Katzendreck”. Therefore, we removed them from the dataset and used them as supplementary elements in the correspondence analysis (CA). The chi-squared test rejected independence between odor characteristics and spatial segments (*p*-value < 0.001). The correspondence analysis revealed that the largest differences in the proportion of odor descriptors were between segments A3+C and B3 (Figure 2b). These segments were placed on the opposite extremes of the first ordination axis which described nearly 68% of the variability in the data. In segments A3 and C, there were mainly odors such as petrol, mineral oil, hydrogen sulfide, agricultural odor, natural gas, or indeterminate odor. On the other hand, in segment B3, there were mainly wood burning and then also coal burning. Between these segments was placed the segment B4 (i.e., the area around Litvínov); there, we often encountered the item, “coal burning”, as well as items characteristic for segments A3 and C. The first ordination axis showed a fundamental difference between the odor pollution recorded in the Czech Republic and Germany. In the case of health symptoms, 56% of records had a “without symptom” item. The most frequently recorded symptoms were headache, cough, and shortness of breath. The “other symptoms” category mainly included these entries: it is difficult to breathe (31.5%); asthmatic attack (18%); burning in throat (14.6%); abdominal pain/nasal mucus (11.2%); metallic taste in the mouth (5.6%); sore throat (4.5%); abdominal pain/diarrhea (3.4%); abdominal pain (2.2%). The chi-squared test rejected the independence between physical symptoms and odor characteristics (*p*-value < 0.1%). We also excluded some categories due to their low theoretical frequencies, and they were used in CA as supplementary elements. According to the CA analysis, the item “without symptom” is mainly associated with a “petrol, mineral oil” item (Figure 2c). The ordination along the first axis (80.5% of the variability) was mostly affected by items “without symptoms” and “shortness of breath”. The item “nausea” (with major effect on the second ordination axis) was mostly associated with “hydrogen sulfide”. Other symptoms were not significantly associated with any particular odor characteristic.

### 3.2. Canister Hydrocarbon Sampling

**Figure 3:**
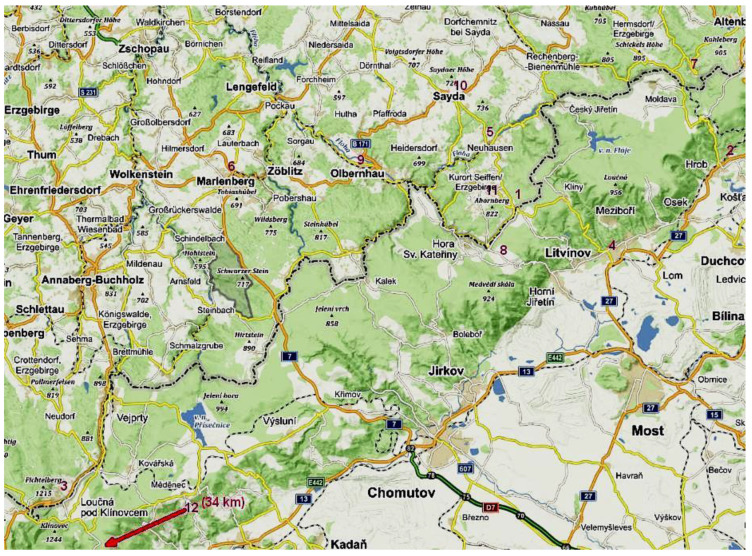
Canister sampling sites. Numbers in red refer to individual sites: 1—Deutscheinsiedel, 2—Háj u Duchcova, 3—Kühnhaide, 4—Litvínov, 5—Neuhausen, 6—Marienberg, 7—Neurehefeld, 8—Nová Ves v Horách, 9—Olbernhau, 10—Sayda, 11—Seiffen, and 12—Vřesová. (Background map: www.mapy.cz (accessed on 5 December 2021 14:19)).

A total of 50 evacuated canister samples were collected in Deutscheinsiedel, Háj u Duchcova, Kühnhaide, Litvínov, Marienberg, Neuhausen, Neurehefeld, Nová Ves v Horách, Olbernhau, Sayda, Seiffen, and Vřesová. The most odor episodes registered and samples collected were in Seiffen (17) and in Háj u Duchcova (9). Figure 3 shows the locations of the twelve canister sampling sites.

Canisters were analyzed by TD-GC-MS; OAV values were calculated based on the results of the chemical analysis. OAV values ranged between 0 ou and 59.75 ou. Results are shown in Table S4 in the Supplementary Materials. Some of the volunteers’ comments, such as, “very strong odor”, did not relate very well with the chemical analyses (e.g., Háj u Duchcova (Site #2 in map) samples 180,111 and 181,030, as seen in Table S4). In these samples, only traces of organic compounds were found by chemical analyses and OVA values were 0.01 and 0.09. The possible explanation is that human response to odor may be based on compounds that were not detected by GC-MS, or odors in mixtures may have been enhanced (or suppressed) in term of perception [40].

Octanal was identified as the main odorant, contributing about 71.7% to the OAV value in samples that exhibited OAV value above 20 ou in Háj u Duchcova. Most of the episodes shown in Table S4 could not be associated with any identifiable source. These unknown sources in Háj u Duchcova are probably situated to the SW of the sampling site as this was the wind direction at the time odors were detected by residents. Compounds measured during odor episodes were mainly aliphatic hydrocarbons, aromatic hydrocarbons, and carbonyls. These compounds may originate from many sources such as biomass burning, cooking, traffic, petrochemical production, coal combustion, biogenic VOC emissions, manure slurry applied as fertilizer, and livestock production systems [10,41,42,43,44,45,46,47].

Seiffen was another location where many odorous episodes were detected and almost all of them were registered when winds were from the SE. Many volatile organic compounds were qualitatively identified and quantitatively determined in these canister samples including odorous compounds such as aromatic hydrocarbons, acetic acid esters, and carbonyls. It was clear that the source cannot be far from the sampling site and the VOCs’ source profile resembled wood furniture coating [3], perhaps originating from a nearby furniture manufacturing plant located to the SE or from similar sources in that general area. Perhalogenated chlorofluorocarbons (CFC) were identified in seven samples in Seiffen, and at high concentrations up to 100 ppb determined using 1,1,2-trichloro-1,2,2-trifluoro ethane as a quantitative standard. Chlorofluorocarbons were identified in landfill gas at waste disposal facilities [47]; on the other hand, CFCs may be added as a foam agent to plastic material [48], and our hypothesis is that probably plastic material was combusted in this manufacture. In Seiffen, 2-propenenitrile was also identified in concentrations ranging from 1.23 to 4.35 ppb and we suppose that also ACN polymers were also combusted in this region. The presence of these substances is surprising in ambient air and outside the scope of European legislation.

Similar compounds were identified in samples from Deutscheinsiedel, Kühnhaide, Litvínov Neurehefeld, and Nová Ves v Horách, see Table S4. These results are supported by measurements in the vicinity of the German–Czech border where garbage combustion is widespread and up to 4% of aerosol has origin in garbage combustion in local heating sources [17]. This result is consistent with a source apportionment study (Pinto et al., 2001) [49], which found that burning garbage was a major PM source in Teplice and in surrounding areas in Northwestern Bohemia. Many aliphatic and aromatic hydrocarbons identified in Lom u Mostu, namely n-pentane, benzene, n-heptane, toluene, and octane are the important emissions from the petrochemical industry [41].

### 3.3. Passive Hydrocarbon Sampling

Using passive samplers, 36 VOC samples were collected at the background sites, 18 samples in Lom u Mostu and 18 in Deutschneudorf (DND). Hydrocarbons 2-methylbutane, pentane, heptane, benzene, toluene, ethylbenzene, styrene, m + p xylene, methyl- cyclopentane, methylcyclohexane, and tetrachloroethylene were identified and quantitatively determined in all samples.

Mean concentrations (ppb) are summarized in Table 3.

Many other analytes were tentatively identified in the collected samples and were calculated using toluene as a reference compound in the concentration range from 10^−3^ to 10^−1^ ppb. A list of semi-quantitatively determined analytes at both sampling sites is available online in Table S3 in the Supplementary Materials.

All concentrations were below the OT value for the measured compounds except for butyric acid whose concentration was 0.63 ppb (calculated as butyric acid/toluene) in LOM, vs. the OT for this compound of 0.19 ppb [35].

The impact of industrial and other sources was estimated in our study by analyzing the benzene to toluene ratio (B/T). A ratio close to 0.6 suggests vehicular emissions as the main source of VOCs; ratios ≤ 0.2 are likely influenced by industrial emissions as toluene is used in many industrial applications. Higher emissions of benzene with respect to toluene with B/T ratio > 1 suggests that the main source responsible for the emissions of the VOCs is possibly biofuel or coal burning [43,50]. Coal burning ratios B/T for French coal burned in power plants is 0.86 [51] and for Czech brown coal burning in a heating plant is 1.51 [52]. Measured B to T ratios are summarized in Table 4.

Relatively high concentrations of benzene were measured in DND with B/T = 2.08. Concentrations of aromatic hydrocarbons—benzene, toluene, m + p-xylene, ethylbenzene were well correlated (R^2^ = 0.936) with published data for pine combustion [53]. These results are in good agreement with measurements in German–Czech border region that soft wood combustion is an important source of aerosol in this region [17].

2-methyl butane is considered as a vehicular emission marker and the DND air shed is probably influenced by transported emissions such as 2-methyl butane, methyl cyclopentane, and methylcyclohexane ratios to toluene are in good agreement with data published for traffic (2-methylbutane 0.68, methylcyclopentane 0.22, and methylcyclohexane 0.08) [54].

Small quantities of tetrachloroethylene are emitted by coal-fired power plants [51] with a ratio to toluene of 0.55. Data in Table 3 indicate that coal combustion is probably another important source of VOCs in this region. Towns and villages situated at high elevations on the Ore Mountains (e.g., DND) are more likely to be influenced by power plant emissions than are sites at lower elevations (e.g., LOM) due to the height of power plant stacks in the foothills of the mountains.

### 3.4. Passive Carbonyl Sampling

Eight-day sampling periods were used; 14 samples were collected in LOM and DND, and 11 samples were collected in JER. Formaldehyde was the most abundant carbonyl in all samples and accounted for 22.2–22.9% of the total ambient air carbonyl concentrations.

Glutaraldehyde, isovaleraldehyde, 2-butanone, dimethylbenzaldehyde, and heptanal were not detected in ambient air samples. O-tolulaldehyde (0.05 ppb), p-tolulaldehyde (0.04 ppb), and octanal (0.33 ppb) were determined in one sample in LOM, and hexaldehyde (0.18 ppb) in one sample in JER. Crotonaldehyde was identified in two samples from LOM and in one sample from DND, and its concentration was close to the quantification limit, 0.1 ppb.

Total mean concentrations of carbonyls were in the range of 2.88–3.06 ppb and are comparable with concentrations measured in an urban (Helsinki) and a remote forested environment in Finland [55,56]. Concentrations of formaldehyde and other aldehydes are expected to be significantly higher in summer as atmospheric photooxidation of hydrocarbons during summer is an important secondary source of carbonyls and involves reactions of ozone, OH, and NO_3_ radicals with organic compounds that are associated with air pollution [57].

Formaldehyde to acetaldehyde (C_1_/C_2_) ratios usually varied from 1 to 2 in urban area and higher values were measured in forested areas, so the ratios can be used as anthropogenic source of formaldehyde. C_1_/C_2_ ratios in the present study ranged from 2.83 to 3.56 and are in agreement with ratios found in Finland and Guangzhou [55,56,58]. Acetaldehyde to propionaldehyde ratio can be used also as a measure of the presence of biogenic sources as propionaldehyde is associated with anthropogenic, mainly industrial, emissions only. C_2_/C_3_ ratios in our study ranged from 1.06 to 1.50 suggesting the possible impact of industrial sources.

Arithmetic means and range of concentrations at sites LOM, DND, and JER together with data reported from previous studies are listed in Table 5.

The sum of C_4_–C_10_ carbonyls at the sampling sites was relatively high compared with C_1_–C_3_ aldehydes, and their ratio to the C_1_–C_3_ aldehydes was in the range of 1.03 to 1.06 and was higher than the value in studies [55,56,58] shown in Table 5. The most abundant high molecular weight of carbonyls butyraldehyde, valeraldehyde, nonanal, and decanal accounted for 35.6–41.3% of the total carbonyl concentrations. This agrees with results indicating that these compounds are ubiquitous in the atmospheric environment, and that direct emissions from plants appears to be a major source of these components in some urban, suburban, and forested areas. In natural environments, nonanaldehyde was also found to be one of the most abundant components where vegetation was growing [59].

### 3.5. Odorous Emission from Cooking Oil Processing

A cooking oil processing plant is situated in the center of the town, Ústí nad Labem-Střekov; many complaints were registered at the local District Office. During the processing of edible oil, many procedures that could lead to odorous emissions including deodorization are used. During the deodorization process, numerous odorous substances such as aldehydes, ketones, hydrocarbons, furans, and terpenes are separated from the oil by distillation. Aliphatic carbonyls (acetaldehyde, acetone, propionaldehyde, 2-butanone, butyraldehyde, benzaldehyde, valeraldehyde, hexaldehyde, heptaldehyde, octaldehyde, nonanaldehyde, decyl aldehyde, 2-heptenal, 2-octenal, 2-nonanal, 2,4-nonadienal, and 2,4-decadienal) are considered as major contributors to undesirable odors from oil processing plants [60,61].

Two monitoring sites on opposite sides and close to the plant were chosen for sampling carbonyls using Radiello^®^ passive samplers. Samples were collected for ten days by volunteers whose task was to monitor and record odors in the environment. Wind speeds and directions often changed during the 10 days of sampling. Odor intensity ranked from 1 to 3 for every odor episode: 1—weak odor, 2—strong odor, and 3—extremely strong odor. Four samples were collected and the weighted average (W) for every sample was calculated according to Equation (5):

W = (I × t) Σt(5)

where I = intensity values from 1 to 3, t = registered time for every episode, and Σt = total time for registered odors.

Samples were analyzed in laboratory and concentrations of carbonyls and odor threshold values (OT) are shown in Table 6.

Calculated values (OAV) were in excellent agreement with perceived smell W (R^2^ = 0.93) and are expressed by Equation (6):

OAV = 21.307 × W + 28.05(6)

## 4. Health Risk Assessment

To assess possible health effects of inhaled compounds, the US EPA Health Risk Assessment Approach was applied as shown below:

1. Hazard identification and data evaluation;
2. Exposure assessment;
3. Dose-response assessment;
4. Risk characterization.

The methodology for assessing cancer risks, non-cancer effects, and related uncertainties has been described [62,63], and this methodology was used for assessing the health effects for many of the compounds, including odorants, that were measured. Equation (7) was used for net intake:

(7) Intake=(C × IR × EF × ED)/(BW × AT)

where C = concentration of VOC in ambient air, IR = intake ratio, EF = exposure frequency, ED = exposure duration, BW = weight and AT = average time of exposure.

Exposure concentration (C) instead of “intake” was used for the calculation of the Hazard Index (HI). Cumulative exposure and risk assessment generally assume exposure paths from more than one medium. Our evaluation focused only on airborne exposure to organic substances during odor episodes.

We based our calculation of HI for mixtures of substances on similarity of the endpoints of species in the group of substances and additivity of the effects. Published reference concentrations [64] were used for calculating HI in Equation (8):

HI = Intake/reference concentration.(8)

And for calculation of HI for the entire mixture, Equation (9) was used:

HI_m_ = Σ HI_i_, i =1,n (9)

where HI_m_ = Hazard Index for the whole mixture of aliphatic and aromatic hydrocarbons and HI_i_ = Hazard Index calculated for the ith component.

We considered chronic exposure during odor episodes in the winter months for three years. A total of 491 episodes were described: 285 in Germany and 206 in the Czech Republic. We assumed that the inhabitants lived in the same location for 40 years. On the Czech side, there were approximately 2500 inhabitants in thirteen municipalities who were likely exposed; the total number of people exposed in Germany is unknown.

Symptoms described by residents were not objectified by medical examination. Health statistics that might have indicated the incidence of specific diseases were not available.

For carcinogenicity assessment, the concentrations of carcinogenic compounds were used with Inhalation Unit Risk values to derive an estimate of the potential Incremental Lifetime Cancer Risk (ILCR) associated with that exposure [63,65]. The ILCR was calculated according to Equation (10):

(10) $$\mathrm{ILCR}=\mathrm{Exposure}\;(\mu g/m^{3})\times \;\mathrm{Inhalation}\;\mathrm{Unit}\;\mathrm{Risk}$$

We considered the load of inhaled organic substances to be chronic. Exposure time was shortened for 5 weeks of holiday spent outside the area.

### 4.1. Risk Assessment

German inhabitants reported noxious odors on 16% of days in the study period and Czech inhabitants reported odors on 12.1% of days. These values were used for the exposure assessment.

To assess health risks, we divided analyzed substances into a complex mixture of aliphatic and aromatic hydrocarbons [64], and we took into account the analyzed ethers, ketones, alcohols, halogenated hydrocarbons, acids, aldehydes, esters, terpenes, uniquely analyzed organic nitrogen, and sulfur compounds. The complex mixture was divided into further fractions: aliphatic fraction C_5_–C_8_, aliphatic fraction C_9_–C_16_, aromatic fraction C_6_–C_8_ (benzene, ethylbenzene, toluene, styrene, xylenes), aromatic fraction C_9_–C_16_ (High Molecular Weight Aromatic Naphtha).

An overview of HI values for different classes of compounds for Czechia and Germany is given in Table 7. HI values were calculated for every canister sample and are given in Table S4 in the Supplementary Materials. As can be seen from Table 7, the mean HI for all compound classes was less than one, with generally lower values on the Czech than on the German side of the border. However, individual values ranged from <0.001 to 3.96 in Saxony. This overall maximum value was recorded in Neuhausen on 4.3.2018. Main contributors to this overall maximum value were: aromatic Naphtha (HI 1.3), xylenes (HI 0.76), Low carbon Range Aliphatic Fraction (C_5_–C_8_) (HI 0.8), and benzene (HI 0.4). These species are mainly associated with petroleum processing and gasoline. For C_3_–C_4_ hydrocarbons, ketones, and ethers, the risk of chronic nervous system and respiratory tract impairment associated with chronic inhalation of gaseous hydrocarbons (propane, butane, isobutane), ketones, and ethers, expressed by HI, never exceeded one. HI values in the Czech Republic were generally one or more orders of magnitude lower than in Germany.

Chlorinated hydrocarbons and chlorofluorocarbons possess many local as well as systemic toxic effects; the most serious include carcinogenicity and mutagenicity, effects on the nervous system, and injury to vital organs, particularly the liver. Despite the relative chemical simplicity of the group, the toxic effects vary greatly, and the relation between structure and effect is often not clear [66]. According to our estimate these compounds exhibited relatively low risk with mean HI values < 1. However, in Neurehefeld, one canister air sample exhibited an HI value of 1.14. In the group of ten chlorofluorocarbons, we could evaluate only two compounds (1,2-dichloro-1,1,2,2-tetrafluoro-ethane, 1,1,2-trichloro-1,2,2-trifluoro-ethane) that had occupational exposure medical limits [67]. Chlorinated and chlorofluorinated substances were found in higher concentrations on the German side.

Aldehydes and acids and their esters are highly irritating to the respiratory tract and mucous membranes exposed by inhalation. Ten aldehydes and 13 alcohols were identified in air and were found not to pose a significant risk at the concentrations measured. Alcohols and aldehydes were found on the German side in low concentrations; only heptanal exhibited a Hazard Index higher than one (HI 1.08) in Seiffen.

Similarly to the aforementioned, six acids were found; in three cases, we were able to evaluate the Hazard Index from existing reference values (acetic acid, formic acid, and methyl propanoic acid); the highest HI = 0.9 belonged to formic acid, which was identified in the air only once in Háj u Duchcova. Esters contributed to HI up to a maximum of 0.5 in two samples in Olbernhau and Seiffen.

Terpenes (limonene and pinenes) were not associated with any significant health risk as can be seen from Table 7. HI values for dimethyl sulfoxide were determined in Neuhausen (HI 0.01), and pyridine in Háj u Duchcova (HI 0.04). 2-propenenitrile was repeatedly identified in Seiffen and the Hazard Index was relatively high with a maximum value = 1.25.

HI values for compounds sampled by volunteers in Seiffen are shown in Table 8. It can readily be seen that HI for nitriles are the highest observed in this study. High maximum HI values were also found for several other compound classes.

Values for OAVs in Seiffen were compared with HI values in Table S4 of the Supplementary Materials. Two-tailed Spearman’s correlation coefficient (r_s_ = 0.87) indicated relatively good agreement in ranks between the two variables.

### 4.2. Carcinogenic Risk Assessment

Twelve of the identified compounds in odor episodes: benzene, isoprene, ethylbenzene, naphthalene, 1,4-dioxane, trichloromethane, tetrachloroethylene, 2-propenenitrile, styrene, methylene chloride, and acrylonitrile are classified as carcinogens. Benzene is a proven human carcinogen and is classified in group I according to IARC; the other compounds are classified as suspected human carcinogens in group II B resp. II A. components [68].

The dose–effect relationship was obtained from the EPA IRIS database [65] and MRL ATSDR [69] from published reference concentrations of the National Institute of Public Health and from occupational health prescriptions for both Czech [70] and OSHA/NIOSH [67] sources (both are identical). To estimate the carcinogenic risk, we use the risk units published in the EPA IRIS database for benzene, 1,4-dioxane, trichloromethane, tetrachlorethylene, methylene chloride [65] for ethylbenzene, and naphthalene from OEHHA California Office for Environmental Hazard Assessment [71,72].

The results are summarized in Table 9 and Table 10.

There are carcinogens whose effects are thought to be threshold-free and which we should always be concerned about. Benzene concentrations in four cases reached tens of ppb. However, canister samples only lasted several minutes and so were not necessarily representative of chronic exposures. The highest carcinogenic risk was associated with the inhalation of 2-propenenitrile in Seiffen, the substance was detected repeatedly (Supplementary Materials Table S4) and always above an ILCR level of 10^−5^. The proven carcinogen benzene yielded ILCR values for the local population of >10^−6^, and in one sample, exceeded 10^−5^; trichloromethane in one case was associated with a risk of 10^−5^. All other pollutants were associated with lower carcinogenic health risk.

### 4.3. Hazard Indexes and Cancer Risk near an Oil Processing Plant in Ústí nad Labem

Hazard Index and cancer risk are summarized in Table 11 and Table 12.

### 4.4. Uncertainty Discussion

Uncertainty is associated with the use of reference values. In the case of petroleum hydrocarbon fractions, we performed a combined evaluation of a mixture of chemicals using reference concentrations from the US EPA IRIS database [65], but also recommended reference values for aliphatic fractions C_5_–C_8_, C_9_–C_16_, and similar aromatic fractions with the same number of carbons [64]. Thus, we evaluated the whole group of hydrocarbons from the fractions. For other hydrocarbon fractions, we did not proceed in this way and used only reference values, or recalculated reference values from occupational medical PELs (Czech and OSHA/NIOSH [16,67]).

The large spectrum of monitored compounds and the relatively low frequency of sampling did not permit a rigorous statistical evaluation, which can only be performed using a much larger data set. Our health risk assessment is associated with high degree of uncertainty. This applies both to the assessment of the chronic non-carcinogenic effects and to the assessment of the carcinogenic risk.

Uncertainty is also high for evaluating long-term exposures. Detected substances were often at background levels, others corresponded to small-scale production using chlorine-containing and non-chlorine-based solvents, e.g., processing plastics or working with acrylic synthetic paints. Assuming the concentrations measured represent chronic exposure levels, there would appear to be a very small inhalation risk of non-carcinogenic effects.

We did not calculate an overall Hazard Index by simply adding the values of all individual substances shown in Table 7, as the pathophysiological and toxic effects of, e.g., acids and aldehydes are different from those of hydrocarbons. However, this does not necessarily mean that synergies between different compounds or classes of compounds do not exist. Similar considerations apply to carcinogenic effects, which is why we left the final evaluation at the level of individual substances as shown in Table 8.

## 5. Summary and Conclusions

A wide variety of VOCs were determined using canister sampling and passive sampling including: aliphatic and aromatic hydrocarbons; ketones, esters; halogenated hydrocarbons; aldehydes; alcohols; terpenes, and nitriles. The most malodorous compounds were: heptanal, pentanal, butyl-ester, and acetic acid. The most unpleasantly smelling compound with the highest Hazard Index was acetic acid. The riskiest substances were (carcinogenic) benzene, tetrachloroethylene, naphthalene, 2-propenenitrile, and heptanal.

During the winter periods from January 2017 to December 2018, transport of high levels of air pollutants across the German–Czech border were not analytically confirmed, indicating that odorous compounds were most likely emitted from nearby local sources. Two such sources of odorous compounds were identified: an edible oil processing plant in Ústí nad Labem (CR) and furniture production in Seiffen (DE). Odors recorded by volunteers close to the oil processing plant were well correlated with analytical results for carbonyls and calculated OAV values (R^2^ = 0.93). Most complaints about odorous compounds were registered in Seiffen. Spearman’s correlation coefficient, r_s_ = 0.87, was calculated for OAV and HI values in Seiffen, indicating relatively good agreement in ranks between the two variables.

More than 60 VOCs were measured using passive sampling at the relatively remote sampling sites, LOM and DND. The calculated ratio of VOCs to toluene indicated that wood combustion could be an important source of VOCs in DND, and that coal combustion in coal-fired power plants and traffic are probably other sources of VOCs in this region. Locations at higher elevations in the Ore Mountains, such as DND, are more likely to be influenced by emissions from power plants, given the height of the power plant stacks located in the foothills, than are locations at lower elevations, such as LOM.

Short-term health risks associated with noxious odors described here are likely small, however, further sampling is needed to better estimate overall, long-term health risks. Even though levels of sampled substances were not associated with acute effects at any site, long-term exposures to selected compounds could be cause for concern for carcinogenicity at some sites. Odors in Seiffen were associated with carcinogenic compounds, 2-propene nitrile, and benzene in canister samples. Although not necessarily representative of long-term exposures to the compounds studied, results such as those presented here suggest that further study is needed to better quantify long-term exposure to potentially harmful compounds, and to either confirm or deny the existence of substantive health risk in the vicinity of the Czech-Saxon border.

## Figures and Tables

**Table 1:** Monitoring sites, station notes, measurements made, and availability of data.

Monitoring Sites	Station Notes	Measurements	Data Availability
Deutschneudorf (DND)-Saxony 50°36′11.75″ N, 13°27′55.68″ E, 767 m a.s.l.	SE of Kurort Seiffen, located right on German–Czech border. Mobile sampling container of Leipzig TROPOS Institute	UFP, BC, and meteorological parameters were measured. Passive sampling of VOCs	
Lom u Mostu (LOM) 50°35′8.757″ N, 13°40′24.305″ E, 257 m a.s.l.	Located on site of demolished village, Libkovice. Sampling container of Czech Hydrometeorological Institute (CHMI)	O_3_, NO, NO_2_, SO_2_, PM_2_._5_, PM_10_ (incl. heavy metals in), BC (PM_1_), UFP. Passive sampling of VOCs	Czech Hydrometeorological Institute web portal [18,19]
Schwartenberg (SCH) 50°39′33.994″ N 13°28′0.002″ E, 787 m a.s.l.	Located on hill near Kurort Seiffen. Sampling container of Staatliche Betriebsgesellschaft für Umwelt und Landwirtschaft of Saxony	O_3_, NO, NO_2_, SO_2_, benzene, PM_10_ (incl. heavy metals in), PAH’s and meteorological parameters	Czech Hydrometeorological Institute web portal [20,21]
Ústí nad Labem (UL) 50°39′39.941″ N, 14°2′35.027″ E, 147 m a.s.l.	Located in the central district of the city UL. Sampling container of Czech Hydrometeorological Institute (CHMI).	O_3_, NO, NO_2_, SO_2_, PM_10_, BC (PM_1_), UFP, benzene, Hg^0^. Passive sampling of VOCs	Czech Hydrometeorological Institute web portal [22,23]
Jeřabina (JER) 50°61′27.167″ N, 13°52′10.558″ E, 777 m a.s.l.	Located in mountain pass between Litvínov region (CR) and Seiffen (DE). Site lacks electrical power	Passive sampling of VOCs	This article

**Table 2:** Percentage of records with (a) characteristic odor or (b) physical symptom item (*n* = 491). For characteristic odors, the percentage reported in either the Czech Republic or Germany are shown. Total percentages for subjective odor descriptors and physical symptoms are greater than 100% because of multiple reporting of odors or symptoms by individuals.

(a) Odor Descriptor	%	% CZ	% DE
petrol, mineral oil	24.8	10.7	89.3
hydrogen sulfide (H_2_S)	21.1	26	74
coal burning	14.2	64.3	35.7
indeterminate character	14.0	2.9	97.1
wood burning	12.8	93.7	6.3
tar, asphalt	12.4	18	82
Katzendreck	9.2	37.8	62.2
natural gas	8.9	36.4	63.6
agricultural odor	8.7	7	93
plastic burning	6.5	65.6	34.4
other odor descriptors and associations with odors	29.5	55.9	44.1
**(b) Subject Physical Symptoms**	**%**		
headache	16.5		
cough	16.1		
shortness of breath	12.6		
nausea	7.1		
smarting eyes, lacrimation	6.5		
faintness, weariness	4.9		
tachycardia	3.3		
vomiting	1.4		
without symptoms	56.4		

**Table 3:** Mean concentrations of VOCs at DND and LOM.

	DND (ppb)	LOM (ppb)
Benzene	0.137	0.222
Toluene	0.066	0.201
Ethylbenzene	0.017	0.031
m + p Xylene	0.033	0.057
Pentane	0.059	0.076
Methylcyclopentane	0.009	0.021
Heptane	0.005	0.016
Methylcyclohexane	0.006	0.014
Tetrachloroethene	0.016	0.017
2-methylbutane	0.031	0.069
Styrene	0.008	0.021

**Table 4:** Ratios of selected VOCs to toluene (ppbv/ppbv) in DND and LOM.

Sampling Site	Benzene	Tetrachloro- ethylene	2-Methylbutane	Methyl- cyclopentane	Methyl- cyclohexane
DND	2.08	0.28	0.47	0.14	0.09
LOM	1.10	0.09	0.34	0.11	0.06

**Table 5:** Arithmetic means and range of carbonyl concentrations (ppb) at three diverse sites based on passive sampling, the Botanic Garden, and a residential area in Guangzhou, China [58], and a remote forested area in Finland [55] and an urban area in Finland, Helsinki [56].

Carbonyl Compound	LOM	DND	JER	Botanic Garden ^1^	Residential Area ^1,2^	Remote Area ^1,3^	Urban Area ^1,3^
formaldehyde	0.68 (0.42–1.07)	0.68 (0.42–1.56)	0.64 (0.34–1.10)	12.38	11.26	0.38	0.24
acetaldehyde	0.24 (0.15–0.57)	0.22 (0.10–0.67)	0.18 (0.07–0.31)	4.25	6.03	0.19	0.07
acetone	0.14 (bd ^4^–0.47)	0.16 (bd–0.43)	0.16 (bd–0.43)	6.72	7.68	0.55	0.36
acrolein	0.23 (bd–0.32)	0.29 (bd–0.38)	0.25 (bd–0.38)			bd	bd
propion– aldehyde	0.16 (bd–0.29)	0.16 (bd–0.28)	0.17 (bd–0.24)	1.15	1.15	0.03	0.03
methacrolein	0.21 (bd–0.3)	0.33 (bd–0.41)	0.16 (bd–0.21}			bd	0.01
butyraldehyde	0.57 (bd–1.32)	0.54 (bd–0.85)	0.49 (bd–0.68)	0.44	0.68	0.02	0.02
valeraldehyde	0.19 (bd–0.44)	0.22 (bd–0.38)	0.27 (bd–0.39)	0.22	0.26	0.02	0.01
benzaldehyde	0.02 (bd–0.03)	0.03 (bd–0.05)	0.03 (bd–0.04)	0.36	1.07	5 × 10^–3^	0.02
nonanal	0.19 (bd–0.53)	0.14 (bd–0.18)	0.16 (bd–0.22)	0.53	0.44	bd	0.02
decanal	0.24 (bd–0.28)	0.19 (bd–0.22)	0.27 (bd–0.27)	0.13	0.06	0.01	0.02
C_1_–C_3_	1.45	1.51	1.40	24.57	26.12	1.15	0.7
C_4_–C_10_	1.52	1.55	1.48	2.47	3.06	0.05	0.09
Total	2.97	3.06	2.88	27.04	29.18	1.20	0.79
Ratio C1/C2	2.83	3.09	3.56	2.91	1.87	2.0	2.18
Ratio C2/C3	1.50	1.38	1.06	3.69	5.23	0.33	0.18

^1^ Conversion from µg·m^−3^ to ppbv is made assuming *p* = 1 atm. T = 298 K. R= 0.082057 L·atm. mol^−1^·k^−1^. ^2^ Samples collected I Guangzhou, China. ^3^ Samples collected in background forest in Finland and Helsinki. ^4^ bd = below detection limit.

**Table 6:** Carbonyl concentrations (ppb) in Ústí nad Labem and odor threshold (OT) values derived from the literature [35].

Analyte/Sample (ppb)	1	2	3	4	OT [35] (ppb.ou ^−1^)
formaldehyde	1.27	2.07	1.75	1.86	500
acetaldehyde	0.76	1.03	0.84	0.89	1.5
acetone	0.70	1.05	0.77	0.96	42,000
propionaldehyde	0.44	0.55	0.40	0.44	3.6
crotonaldehyde	0.11	0.13	0.13	0.14	1.0
methacrolein	0.20	0.33	0.21	0.29	8.5
2-butanone	0.11	0.11	0.11	<0.02	28
butyraldehyde	1.12	1.46	1.04	1.93	0.67
benzaldehyde	0.09	0.11	0.10	0.04	0.18
isovaleraldehyde	0.06	0.06	<0.02	<0.02	0.10
valeraldehyde	0.68	0.81	0.82	0.80	0.41
hexanal	0.39	0.55	0.39	0.46	0.28
heptanal	0.60	0.68	0.33	0.66	0.18
octanal	0.61	0.49	0.48	0.73	0.01
nonanal	0.41	0.89	0.61	0.98	0.34
decanal	0.47	0.84	0.82	0.90	0.40
OAV (ou)	73.7	66.5	60.3	89.4	
W	2.21	2.0	1.38	2.75s	

**Table 7:** Hazard Index (HI) for chronic, non-carcinogenic effects from exposure to VOCs during odor episodes in the vicinity of the German–Czech border.

		HC	C_3_–C_4_	HAL HC	ALD	ALCO	OA	ESTERS	TERP	2-PRCN
DE	mean	0.153	0.041	0.148	0.541	0.028	0.135	0.171	0.001	0.645
	max	3.960	0.158	1.142	1.081	0.190	0.208	0.557	0.006	1.205
	min	0.000	0.002	0.000	0.000	0.001	0.075	0.000	0.000	0.332
CZ	mean	0.022	0.001	0.004	bd	0.002	0.478	0.002	0.003	bd
	max	0.227	0.001	0.023	bd	0.007	0.908	0.003	0.007	bd
	min	0.001	0.000	0.000	bd	0.001	0.049	0.002	0.000	bd

Abbreviations: DE—Germany; CZ—Czech Republic; HC—complex mixtures of aliphatic and aromatic hydrocarbons; C_3_–C_4_—C_3_–C_4_ hydrocarbons, ketones, ethers; HAL HC—halogenated hydrocarbons; ALD—aldehydes; ALCO—alcohols; TERP—terpenes; 2-PRCN—2-propenenitrile; bd—below detection limit.

**Table 8:** Hazard Index of chemical substances sampled by volunteers in Seiffen, 2017–2018.

	AL HC	AR HC	ALCO	OA	ESTERS	HAL HC	TERP	CN	Total
Median *	0.017	0.056	0.086	0.726	0.840	0.090	0.004	3.608	1.840
Max	0.325	0.562	1.484	1.434	3.632	0.588	0.005	8.325	9.486
Min	0.001	0.002	0.011	0.017	0.006	0.007	0.004	2.290	0.002

Abbreviations: AL HC—aliphatic hydrocarbons; AR HC—aromatic hydrocarbons; ALCO—alcohols; OA—organic acids; HAL HC—halogenated hydrocarbons; TERP—terpenes; CN—nitriles. * Due to the small number of samples, the median instead of the average was used.

**Table 9:** Estimated individual lifetime cancer risk (×10^6^) from exposure to carcinogenic compounds during the odor episodes near the Czech–Saxon border.

		Isoprene	Benzene	Ethyl benzene	Naphthalene	1,4-Dioxane	Trichloro- methane	Tetrachloro-ethylene	Methylenechloride	2-Propene- nitrile
DE	mean	0.00612	14.9	7.38	6.32		0.135	0.0978	0.171	50.2
	max		61.8	34.4				0.505		93.7
	min		1.33	0.288				0.0155		25.8
CZ	mean		6.74	3.26		0.161	15.7	0.0437		
	max					0.191		0.185		
	min					0.131		0.00559		

Abbreviations: DE = Germany; CZ = Czech Republic.

**Table 10:** Estimated individual lifetime cancer risk (×10^6^) of chemical substances sampled by volunteers in Seiffen 2017–2018.

	Isoprene	Benzene	Ethyl benzene	Naphtha- lene	Tetrachloro- ethylene	Methylene chloride	2-Propene- nitrile	Total
Median*	0.0061	1.9	1.0	6.3	0.10	0.17	41	50
Max		7.3	1.3		0.51		94	
Min		1.3	0.73		0.077		26	

* Due to the small number of samples, the median instead of the average was used.

**Table 11:** Hazard Index of aldehydes near the oil processing plant in Střekov-Ústí nad Labem monitored between 28 September–8 October and 8–18 October 2018.

Location	FORM	ACET	Acetone	PROP	CROTON	2-Butanone	Total
Purkyňova	0.213	0.182	0.006	0.15	0.35	0.525	1.426
Železničářská	0.23	0.177	0.006	0.126	0.405	0.55	1.493

Abbreviations: FORM = formaldehyde; ACET = acetaldehyde; PROP = propionaldehyde; CROTON = crotonaldehyde.

**Table 12:** Individual lifetime cancer risk (×10^6^) of inhabitants of Střekov-Ústí nad Labem ^1^.

	Purkyňova	Železničářská
Formaldehyde	27	29
Acetaldehyde	3.6	3.5
Total ILCR	31	33

^1^ IUR—IRIS EPA used for calculation.

## Data Availability

Data are available on request.

## Supplementary Materials

The following supporting information can be downloaded at: https://www.mdpi.com/article/10.3390/ijerph19031296/s1, Table S1: Participant demographic characteristics (average age: CZ: 44, DE: 61); Table S2. VOC standards used for external calibration; Table S3. Canister samples, GC-MS analyses, ou values and CAS numbers; Table S4. Hazard Index (HI) for chronic non-carcinogenic effects from exposure to chemicals during the odor episodes near the Czech–German border.
