# Supplementary material for: VOCs and Odor Episodes near the German–Czech Border: Social Participation, Chemical Analyses and Health Risk Assessment
Source: Int J Environ Res Public Health. 2022 Jan 24;19(3):1296. doi: 10.3390/ijerph19031296 (PMC8835392; doi:10.3390/ijerph19031296)
Supplement: Supplementary file 1 [file ijerph-19-01296-s001.zip › ijerph-1519712-supplementary.pdf]

Supplementary Information

Table S1. Participant demographic characteristics (average age: CZ: 44, DE: 61)

		No. (%)
**gender**	male	8 (33)
	female	16 (67)
**age**	20-29	2 (8)
	30-39	3 (13)
	40-49	6 (25)
	50-59	5 (21)
	60-69	6 (25)
	70-79	2 (8)
**nationality**	CZ	14 (58)
	DE	10 (42)

Analytical Methods

Passive samples VOCs analyses by TD-GC-MS.

The sampled cartridge analyses used a thermal desorption system (TD Unity Markes) coupled to gas chromatograph (HP 6890 Agilent). Thermal desorption of VOC was performed in several steps: The sampling tube was desorbed at 300°C and released VOCs were flushed to a trap. The trap was cooled to -10°C and purged with 30 ml.min^{-1} helium flow. It was constituted of two adsorbents Carbotrap C and Carbopack B. In the second step the trap was heated at 290°C allowing the analytes to be split injected (1:40) with helium into the column. The helium flow was maintained at 0.8 ml.min^{-1} and temperature program: 40°C/ 10°C.min^{-1} / 230°C, hold 8 min was used. Adsorbent cartridges need to be conditioned at 350°C before use and residual mass average of the mass blank value for each compound of interest was determined by TD-GC-MS analysis.

Gas chromatograph was equipped with an analytical column DB-624 (60 m x 0,25 mm x 1.4 µm) and a mass spectrometer (MS HP5973) which was operated at full scan from 35 to 300 Daltons. The ionization method was electron impact (EI 70eV). VOCs were identified on their retention times and mass spectra and quantified by external calibration. For a few compounds that lacked authentic standard were tentatively identified and were calculated using toluene as a reference compound as below:

$$C_n = A_n/A_{toluene} \times C_{toluene} \qquad (4)$$

Where:

A_n is the peak area of compound n

$A_{toluene}$ is the peak area of toluene

C_n is the concentration of compound n (ppbv)

$C_{toluene}$ is the concentration of toluene (ppbv) which was quantified by external calibration

Analytes were quantified using a calibration curve, based on the analysis of cartridges loaded and positive blank values subtracted from the analytical results. For each level of calibration 1µl of methanol standard solution or 1L of gas standard diluted in Silco Can canister was used. Detection limits were calculated from the standard deviation of blank values. For compounds for which blank value cannot be measured, the detection limit was estimated from the ratio between signal / noise observed in the analysis of a cartridge loaded with a mass about 0.3 ng.

TD-GC-MS analyses of canister samples.

In the laboratory the canister with sample was pressurized at 0.2 MPa with ultra-pure nitrogen and 2 liter samples were concentrated at 100 ml/min using restrictor on sorption tube Tenax TA+ Carbograph TD-1+ Carboxen 1003 for VOCs analyses. Tenax TA + Sulficarb tubes were used for thiols analyses (Markes). The conditions of TD-GC-MS analyses were the same as described above, but in this case splitless injection was used.

HPLC analyses of carbonyls.

The sampled material was eluted from the cartridges by washing with 2 ml acetonitrile and diluted with 2 ml of ultrapure water and 80 µL of the sample was injected. Analytes were separated on an analytical column Ascentis RP-Amide, 15 cm x 4.6 mm x 3 µm (Supelco) with C_{18} guard column 4 mm x 3 mm (Phenomenex). Gradient elution at flow 1.4 ml/min was used from 100% A (acetonitrile/water 40:60), to 100% B (acetonitrile/water 75:25) and detected by HPLC-UV/VIS detector at 365 nm. A hydrazone standard mixing solution containing formaldehyde, acetaldehyde, acetone, acrolein, propionaldehyde, crotonaldehyde, methacrolein, 2-butanone, butanal, isobutanal, benzaldehyde, cyclohexanone, isovaleraldehyde, valeraldehyde, o-tolualdehyde, m-tolualdehyde, p-tolualdehyde, glutaraldehyde, hexanal, dimethyl benzaldehyde, heptanal, octanal, nonanal, decanal (Supelco, Sigma Aldrich) was used for external calibration. Butyraldehyde and isobutyraldehyde, benzaldehyde and cyclohexanone, p-tolualdehyde and m-tolualdehyde could not be separated on the used analytical column and were reported as butyraldehyde, benzaldehyde and p-tolualdehyde respectively.

Standards used for analysis of other VOCs.

Commercial mixtures of volatile compounds in nitrogen and in methanol and neat standards (dissolved in the laboratory in methanol) were used for calibration. EPA 524 VOC MIX A (0.2 mg/ml methanol, Supelco), Aldehyde standard mix (1 mg/ml methanol, Chromservis), 1,2,3,4-tetramethylbenzene (0.100 mg/ml methanol, Dr. Ehrenstorfer), Ozone precursors – Scott Air (100 ppb/m^3 N_2, Restek), Mercaptans– Scott Air (1 ppm/m^3 N_2, Restek), 1,1,2-trichloro-1,2,2-trifluoro ethane, (Freon 113) (1.0 mg/ml MeOH, Supelco), ethyl acetate, butyl acetate, methyl-isobutyl ketone, 2-butanone, limonene, α-pinene, 1-butanol, 2-pentanone, propionic acid, methyl butyrate, benzoic acid, benzene, ethylbenzene, toluene, m-xylene, p-xylene, o-xylene, styrene, pentane, n-butanal, methylcyclopentane, heptane, methylcyclohexane (Sigma Aldrich). Analytical characteristics for quantitative standards are shown below in Table S2.

Table S2. VOC standards used for external calibration.

**Carbonyls**	**Calibration range ng/l**	**LOQ (ng/l)**	**Uncertainty (%)**
Butanal	1.04-8.33	1.0	8.15
3-methylbutanal	1.04-8.33	1.0	7.64
Pentanal	1.04-8.33	1.0	13.2
Hexanal	1.04-8.33	1.0	14.9
Heptanal	1.04-8.33	1.0	13.0
Benzaldehyde	1.04-8.33	4.0	45.8
Octanal	1.04-8.33	1.0	12.3
Acetophenone	1.04-8.33	20	16.7
Nonanal	1.04-8.33	2.0	15.5
Decanal	1.04-8.33	2.0	17.1
Methyl-isobutyl ketone	1-8.33	1.0	15.0
2-butanone	1-8.33	1.0	15.0
1-butanol	1-8.33	1.0	15.0
2-pentanone	1-8.33	1.0	15.0
**Hydrocarbons**			
2-methylpentane	0.5-8.33	0.5	32.8
Cyclopentane	0.5-8.33	0.5	3.94
3-methylpentane	0.5-8.33	0.5	19.2
1-Hexene	0.5-8.33	0.5	10.1
2,4-dimethylpentane	0.5-8.33	0.5	15.9
Methylcyclopentane	0.5-8.33	0.5	9.3
Cyclohexane	0.5-8.33	0.5	6.7
2-methylhexane	0.5-8.33	0.5	14.9
2,3-dimethylpentane	0.5-8.33	0.5	11.4
3-methylhexane	0.5-8.33	0.5	19.3
Benzene	2-8.33	2.0	30.0
Heptane	0.5-8.33	0.5	16.3
Methylcyclohexane	0.5-8.33	0.5	3.94
2,3,4-trimethylpentane	0.5-8.33	0.5	5.88
2-methylheptane	0.5-8.33	0.5	1.01
3-methylheptane	0.5-8.33	0.5	2.5
Octane	0.5-8.33	0.5	3.72
Toluene	0.5-8.33	0.5	10.3
Ethylbenzene	0.5-8.33	0.5	2.6
Nonane	0.5-8.33	0.5	5.26
p-Xylene	0.5-8.33	0.5	3.82
Styrene	0.5-8.33	0.5	7.85

Isopropyl benzene	0.5-8.33	0.5	2.42
Propylbenzene	0.5-8.33	0.5	2.38
Decane	0.5-8.33	0.5	4.28
1-ethyl-3-methylbenzene	0.5-8.33	0.5	4.9
1-ethyl-4-methylbenzene	0.5-8.33	0.5	6.29
1,3,5-trimethylbenzene	0.5-8.33	0.5	2.42
1-ethyl-2-methylbenzene	0.5-8.33	0.5	3.75
1,2,4-trimethylbenzene	0.5-8.33	0.5	4.87
1,2,3-trimethylbenzene	0.5-8.33	0.5	2.89
1,3-diethylbenzene	0.5-8.33	0.5	3.81
1,4-diethylbenzene	0.5-8.33	0.5	2.42
Undecane	0.5-8.33	05	3.2
**Esters**			
Ethylacetate	1-8.33	1.0	15.0
Butylacetate	1-8.33	1.0	15.0
Methylbutyrate	1-8.33	1.0	15.0
**Terpenes**			
Limonene	1-8.33	1.0	15.0
α-Pinene	1-8.33	1.0	15.0
**Acids**			
Propionic acid	1-8.33	1.0	15.0
Benzoic acid	1-8.33	4.0	15.0
**Halogenated compounds**			
Freon 113	1-8.33	1.0	15.0
**Sulphur compounds**			
Methanethiol	5.2-41.5	2.5	30.0
Ethanethiol	5.2-41.5	2.5	30.0
Dimethylsulfide	5.2-41.5	2.5	30.0
Propanethiol	5.2-41.5	2.5	30.0

S3. Volatile organic compounds identified in LOM and DND

LOM: 2-methylpentane, 3-methylpentane, 2-methylhexane, 3-methylhexane, 2-methylheptane, 3-methylheptane, decane, dodecane, tetradecane, eicosane, 2-methyl-1-propene , 1-butene, 2-butene, 2-methyl-1-pentene, 1-hexene, 1-octene, 2-octene, 4-octene, ethylcyclopentane, 1,3-dimethylcyclohexane, 1,2-dimethylcyclohexane, ethylcyclohexane, 1,1,3-trimethylcyclohexane, decahydronaphthalene, 1,3-dimethylbenzene, 1,3,5-trimethylbenzene, 1,2,3-trimethylbenzene, 1,2,4-trimethylbenzene, 1-ethyl-2-methylbenzene, 1-ethyl-3-methyl benzene, naphthalene, butyl formate, butyric acid, propionic acid, butyl butyrate, ethyl acetate, D-limonene, furan.

DND: 2-methylpentane, 3-methylpentane, tetradecane, 2-methyl-1-propene, 2-methyl-1-pentene, 1-hexene, 1-octene, 2-octene, 1,3,5-cykloheptatriene, 1,3-dimethylbenzene, 1-ethyl-3-methylbenzene, 1-ethyl-2-ethylbenzene, 1,3,5-trimethylbenzene, 1,2,3-trimethylbenzene, 1,2,4-trimethylbenzene, butyl formate, butyric acid, propionic acid, butyl butyrate, ethyl acetate, isopropyl alcohol.

Table S3. Canister samples, GC-MS analyses, ou values and CAS numbers.

Analyte names are in the format generated by NIST 02.

Sampling date	Locality	Compound	CAS	μg/m^3	ppb	ou	Σ ou
12.1.2018	Deutscheinsiedel	Ethyl chloride	000075-00-3	5.26	1.37		0.08
		Ethanol	000064-17-5	40.17	10.49	0.0202	
		Acetic acid, methyl ester	000079-20-9	29.55	7.71	0.0077	
		Ethyl acetate	000141-78-6	53.16	14.51	0.0167	
		Trichloromethane	000067-66-3	0.71	0.18	0	
		Ethane, 1-ethoxy-1-methoxy	010471-14-4	15.65	4.09		
		Methane, diethoxy-	000462-95-3	15.05	3.93		
		Heptane	000148-82-5	4.67	1.12	0.0017	
		Methyl isobutyl ketone	000108-10-1	20.92	5.02	0.0295	
		Toluene	000108-88-3	1.3	0.34	0.001	
		Tetrachloroethylene	000127-18-4	1.35	0.35	0.0005	
		Butane, 1,1,3,4-tetrachloro-1,2,2,3,4,4-hexafluoro-	000423-38-1	93.17	11.96		
		Butane,1,2,4-trichloro-heptafluoro-	000335-45-5	14.84	1.9		
		Ethane, 1,1,2-trichloro-1,2,2-trifluoro-	000076-13-1	146.46	18.8		
10.11.2017	Háj u Duchcova	Pentane, 2,2,4-trimethyl-<br>Toluene	000540-84-1<br>000108-88-3	50.15<br>2.34	13.09<br>0.61	0.0195<br>0.0018	0.11
		Tetrachloroethylene	000127-18-4	0.52	0.14	0.0002	
		5-Hepten-2-one, 6-methyl-	000110-93-0	0.67	0.18		
		Phenol	000108-95-2	1.39	0.36	0.065	
		Ethanol, 2-phenoxy-	000122-99-6	3.23	0.84		
16.11.2017	Háj u Duchcova	Hexane, 3-methyl-	000589-34-4	4.46	1.07	0.0013	0.55
		Formic acid	000064-18-6	2.49	0.65		

		Acetic acid	000064-19-7	11.1	2.9	0.4829	
		Toluene	000108-88-3	3.6	0.94	0.0028	
		Xylene, m+p	000106-42-3	2.65	0.6	0.0146	
		Xylene, o-	000108-38-3	3.01	0.79		
		Phenol	000108-95-2	1.06	0.28	0.0496	
24.11.2017	Háj u Duchcova	Benzene	000071-43-2	9.09	2.8	0.001	0.12
		Cyclohexane, methyl-	000108-87-2	2.53	0.62	0.0041	
		Heptane, 2-methyl-	000592-27-8	0.44	0.11	0.001	
		3-Octene, (Z)-	014850-22-7	0.31	0.08	0.0804	
		Toluene	000108-88-3	5.25	1.37	0.0042	
		Tetrachloroethylene	000127-18-4	0.52	0.14	0.0002	
		Xylene, m+p		4.5	1.02	0.0249	
		1,5-Cyclooctadiene, 1,5-dimethyl-	003760-14-3	1.01	0.26		
		Butane, 1,1,3,4-tetrachloro-1,2,2,3,4,4-hexafluoro-	000423-38-1	0.9	0.24		
		Pentanoic acid, 2,2,4-trimethyl-3-carboxyisopropyl, isobutyl ester	1000140-77-5	4.34	1.13		
26.11.2017	Háj u Duchcova	2-Pentene, (E)-	000646-04-8	2.05	0.53		2.47
		2-Butene, 2-methyl-	000513-35-9	5.85	1.53		
		Pentane, 2-methyl-	000107-83-5	18.15	4.74	0.0007	
		Pentane, 3-methyl-	000096-14-0	15.09	4.21	0.0005	
		1-Pentene, 2-methyl-	000763-29-1	1.51	0.39		
		3-Hexene, (E)-	013269-52-8	0.52	0.14		
		2-Pentene, 2-methyl-	000625-27-4	0.33	0.09		
		Cyclopentane, methyl-	000096-37-7	6.58	1.88	0.0011	
		Cyclohexane	000110-82-7	9.8	2.92	0.0012	
		Ethyl acetate	000141-78-6	1.83	0.5	0	
		Hexane, 3-methyl-	000589-34-4	6.46	1.55	0.0018	
		Benzene	000071-43-2	11.79	3.63	0.0013	
		Heptane	000148-82-5	6.54	1.57	0.0023	
		Cyclohexane, methyl-	000108-87-2	2.33	0.57	0.0038	
		Cyclopentane, ethyl-	001640-89-7	0.72	0.19		
		Heptane, 2-methyl-	000592-27-8	3.51	0.74	0.0067	

		Heptane, 4-methyl-	000589-53-7	0.45	0.12	0.0001	
		Heptane, 3-methyl-	000589-81-1	3.37	0.71	0.0005	
		Methyl isobutyl ketone	000108-10-1	1.52	0.36	0.0021	
		3-Octene	014850-22-7	0.49	0.13		
		Toluene	000108-88-3	63.41	16.55	0.0502	
		Ethylbenzene	000100-41-4	19.73	4.47	0.0263	
		Xylene, m+p		64.93	14.71	0.3588	
		Benzene, propyl-	000103-65-1	5.4	1.08		
		Benzene, 1-ethyl-3-methyl-	000620-14-4	14.64	2.93	0.1628	
		Benzene, 1-ethyl-4-methyl-	000622-96-8	4.2	0.84	0.0101	
		Benzene, 1,3,5-trimethyl-	000108-67-8	2.9	0.58	0.0034	
		Benzene, 1-ethyl-2-methyl-	000611-14-3	5.25	1.05	0.0142	
		Benzene, 1,2,4-trimethyl	000095-63-6	22.34	4.47	0.0373	
		Benzene, 1,2,3-trimethyl	000526-73-8	5.55	1.11		
		Benzene, 1,3-diethyl-	000141-93-5	4.86	0.87	0.0124	
		Benzene, 1,4-diethyl-	000105-05-5	3.85	0.69	1.7692	
		Benzene, 4-ethyl-1,2-dimethyl-	000934-80-5	1.9	0.5		
		Benzene, 1-ethyl-2,3-dimethyl-	000933-98-2	1.06	0.28		
		Benzene, 1,2,4,5-tetramethyl-	000095-93-2	1.03	0.27		
		Ethane, 1,1,2-trichloro-1,2,2-trifluoro-	000076-13-1	6.03	0.77		
3.12.2017	Háj u Duchcova	Toluene	000108-88-3	3.52	0.92	0.003	0
1.1.2018	Háj u Duchcova	Heptane	000148-82-5	2.79	0.67	0.001	2.23
		Toluene	000108-88-3	4.21	1.1	0.0033	
		Tetrachloroethylene	000127-18-4	1.04	0.27	0.0004	
		Hexanal	000124-19-6	1.1	0.264	0. 94	
		Xylene, m+p		4.81	1.09	0.0266	
		D-Limonene	005989-27-5	5.23	0.92	0.0243	
		Nonanal	000124-19-6	2.5	0.42	1.2432	
11.1.2018	Háj u Duchcova	Butane, 2-methyl-	000078-78-4	3.86	1.01	0.0008	0.01
		1-Hexene	000592-41-6	3.88	1.11	0.0079	

		Trichloromethane	000067-66-3	1.32	0.35	0.0001	
		Cyclohexane, 1,2-dimethyl-, trans-	006876-23-9	1.79	0.47		
		Toluene	000108-88-3	5.17	1.35	0.0041	
		Cyclohexene, 1-methyl-4-(1-methylethenyl)-, (S)-	005989-54-8	1.17	0.31		
7.3.2018	Háj u Duchcova	2-Butanone	000078-93-3	3.38	1.13	0.0403	3.62
		Methyl isobutyl ketone	000108-10-1	1.63	0.39	0.0023	
		Pyridine	000110-86-1	2.2	0.57	0.0091	
		Toluene	000108-88-3	2.26	0.59	0.0018	
		Tetrachloroethylene	000127-18-4	0.52	0.14	0.0002	
		Hexanal	000066-25-1	1.21	0.29	1.0376	
		Xylene, m+p		2.96	0.67	0.0163	
		Nonanal	000124-19-6	3.05	0.52	1.5167	
		Decanal	000112-31-2	2.58	0.4	0.9927	
30.10.2018	Háj u Duchcova	2-Butanone	000078-93-3	1.88	0.63	0.0224	0.09
		Heptane, 2,5-dimethyl-	002216-30-0	2.6	0.68		
		Toluene	000108-88-3	3.26	0.85	0.0026	
		1,3-Dioxane, 2,4-dimethyl-	000766-20-1	0.77	0.2		
		Tetrachloroethylene	000127-18-4	2.55	0.67	0.0009	
		Xylene, m+p	000106-42-3	5.3	1.2	0.0293	
		Styrene	000100-42-5	3.29	0.76	0.0217	
		1,3,5,7-Cyclooctatetraene	000629-20-9	3	0.78		
		1R-α-Pinene	007785-70-8	1.05	0.19	0.0103	
		Benzene, 1,2,4-trimethyl-	000095-63-6	2.03	0.53	0.0044	
8.2.2017	Kühnheide	1,4-Pentadiene	000591-93-5	1.55	0.41		1.93
		Toluene	000108-88-3	1.47	0.38	0.0012	
		Tetrachloroethylene	000127-18-4	2.54	0.37	0.0005	
		Styrene	000100-42-5	5.04	1.16	0.0333	
		Limonene	000138-86-3	0.9	0.16	0.0042	
		Decanal	000112-31-2	4.91	0.76	1.8899	
		Ethanol	000064-17-5	205.28	53.58	0.103	
		Methane, dimethoxy-	000109-87-5	18.55	4.84		
		Ethane, 1,1-dimethoxy-	000534-15-6	6.72	1.75		
		Ethyl acetate	000141-78-6	11.46	3.13	0.0036	

		Ethane, 1-ethoxy-1-methoxy-	010471-14-4	19.9	5.19		
		Methane, diethoxy-	000462-95-3	5.36	1.4		
		Benzene	000071-43-2	3.44	1.06	0.0004	
		Ethane, 1,1-diethoxy-	000105-57-7	6.55	1.71		
		Methyl isobutyl ketone	000108-10-1	25.36	6.09	0.0358	
		Toluene	000108-88-3	2.45	0.64	0.0019	
		Xylene, m+p		2.47	0.56	0.0137	
		Styrene	000100-42-5	1.56	0.36	0.0103	
		Butane, 1,1,3,4-tetrachloro-1,2,2,3,4,4-hexafluoro-	000423-38-1	13.97	1.79		
		Butane,1,2,4-trichloro-heptafluoro-	000335-45-5	5.97	0.77		
		Ethane, 1,1,2-trichloro-1,2,2-trifluoro-	000076-13-1	28.72	3.69		
4.2.2017	Litvínov	Toluene	000108-88-3	1.79	0.47	0.001415813	2.14
		Propylene glycol	000057-55-6	1.38	0.36		
		Isopropyl alcohol	000067-63-0	15.24	3.98	0.000152986	
		Acetophenone	000098-86-2	1	0.2	0.100083229	
		Decanal	000112-31-2	5.3	0.82	2.04009283	
		Ethanol, 2-phenoxy-	000122-99-6	2.92	0.76		
7.11.2018	Litvínov	2-butanon	000071-36-3	1.96	0.65	0.02	0.02
		1,3-Hexadien-5-yne	010420-90-3	11.24	2.93		
		Toluene	000108-88-3	5.06	1.32	0	
		Benzene propanoic acid, octyl ester	037826-57-6	2.55	0.66		
16.11.2017	Litvínov	Toluene	000108-88-3	4.48	1.17	0.0035	0.02
		Tetrachloroethylene	000127-18-4	0.26	0.07	0.0001	
		Xylene, m+p		2.78	0.63	0.0154	
		1,3,6-Octatriene, 3,7-dimethyl-, (Z)-	003338-55-4	0.67	0.17		

		Benzene, (2-methyl-1-propenyl)-	000768-49-0	2.13	0.55		
16.11.2018 One hour later	Litvínov	Ethanol	000064-17-5	60.18	15.71	0.0302	24.3
		2-Butanone	000078-93-3	7.65	2.55	0.0911	
		2-Butanol	000078-92-2	2.06	0.54	0.0489	
		1-Butanol	000071-36-3	4.01	1.3	0.0342	
		Toluene	000108-88-3	5.36	1.4	0.0042	
		Hexanal	000066-25-1	1.38	0.33	1.1834	
		Xylene, m+p		3.62	0.82	0.02	
		Octanal	000124-13-0	1.22	0.23	22.8851	
		Butane, 1,2,3,4-tetrachloro-1,1,2,3,4,4-hexafluoro-	000375-45-1	8.78	1.13		
		Ethane, 1,1,2-trichloro-1,2,2-trifluoro-	000076-13-1	3.37	0.43		
8.2.2017	Marienberg	Pentane, 2-methyl-	000107-83-5	2.72	0.76	0.0001	26.09
		Pentane, 3-methyl-	000096-14-0	7.38	2.06	0.0002	
		Hexane, 2-methyl-	000591-76-4	2.17	0.52	0.0012	
		Hexane, 3-methyl-	000589-34-4	2.58	0.62	0.0007	
		Toluene	000108-88-3	2.68	0.7	0.0021	
		Heptane, 3-methylene-	001632-16-2	1.867	0.487	0.0003	
		Hexanal	000066-25-1	1.46	0.35	1.2522	
		Tetrachloroethylene	000127-18-4	1.68	0.24	0.0003	
		1-Octene, 4-methyl-	013151-12-7	0.655	0.171		
		4-Nonene	002198-23-4	0.53	0.138		
		Styrene	000100-42-5	1.94	0.45	0.0128	
		Nonane	000111-84-2	1.39	0.26	0.0001	
		α-Pinene	000080-56-8	1.24	0.22	0.0122	
		Octanal	000124-13-0	1.06	0.2	19.8835	
		Limonene	000138-86-3	1.68	0.3	0.0078	
		Acetophenone	000098-86-2	1.34	0.27	0.1341	
		Undecane	001120-21-4	1.43	0.22	0.0003	
		Nonanal	000124-19-6	3.62	0.61	1.8002	
		Dodecane	000112-40-3	1.44	0.16	0.0015	
		Decanal	000112-31-2	7.74	1.19	2.9793	
23.1.2018	Neuhausen	Toluene	000108-88-3	2.53	0.66	0.002	0.06

		Xylene, m+p		3.18	0.72	0.0176	
		Styrene	000100-42-5	5.89	1.36	0.0389	
		D-Limonene	005989-27-5	1.32	0.23	0.0061	
		Ethane, 1,1,2-trichloro-1,2,2-trifluoro-	000076-13-1	9.9	1.27		
		Butane, 1,1,3,4-tetrachloro-1,2,2,3,4,4-hexafluoro-	000423-38-1	9.5	1.22		
10.3.2018	Neuhausen	Propane	000074-98-6	111.86	29.2	0	43.49
		Isobutane	000075-28-5	144.61	37.75		
		Butane	000106-97-8	360.92	94.21	0.0001	
		Ethanol	000064-17-5	299.89	78.28	0.1505	
		Toluene	000108-88-3	2.38	0.62	0.0019	
		Tetrachloroethylene	000127-18-4	0.72	0.19	0.0002	
		Hexanal	000066-25-1	1.42	0.34	1.2177	
		Dimethyl sulfoxide	000067-68-5	0.46	0.12		
		1R-α-Pinene	007785-70-8	2.26	0.4	0.0222	
		Decane	000124-18-5	1.33	0.35	0.0006	
		β-Pinene	000127-91-3	1.11	0.29	0.0088	
		Octanal	000124-13-0	2.02	0.38	37.8911	
		D-Limonene	005989-27-5	8.54	1.51	0.0397	
		Eucalyptol	000470-82-6	6.81	1.78		
		Nonanal	000124-19-6	3.84	0.65	1.9096	
		Decanal	000112-31-2	5.84	0.9	2.247	
24.3.2018	Neuhausen	Butane, 2-methyl-	000078-78-4	608.15	158.74		10.16
		Ethanol	000064-17-5	487.74	127.31	0.2448	
		2-Pentene	000109-68-2	177.32	46.28	0.4628	
		Cyclopropane, 1,2-dimethyl-, cis-	000930-18-7	273.95	71.51		
		Pentane, 2-methyl-	000107-83-5	189.92	53	0.0076	
		Pentane, 3-methyl-	000096-14-0	354.68	98.98	0.0111	
		Hexane	000110-54-3	228.83	63.86	0.0426	
		2-Hexene	000592-43-8	94.48	24.66	0.1761	
		2-Pentene, 3-methyl-, (Z)-	000922-62-3	26.3	6.86		
		2-Hexene, (Z)-	007688-21-3	25.58	6.68	0.0477	

		2-Pentene, 3-methyl-	000922-61-2	57.72	15.07			
		Cyclopentane, methyl-	000096-37-7	212.41	60.7	0.0357		
		Cyclopentene, 1-methyl-	000693-89-0	48.93	12.77			
		Hexane, 2-methyl-	000591-76-4	109.16	26.2	0.0624		
		Hexane, 3-methyl-	000589-34-4	101.07	24.26	0.0289		
		Benzene	000071-43-2	95.75	29.48	0.0109		
		Heptane	000142-82-5	120.5	28.92	0.0432		
		3-Methyl-3-hexene	003404-65-7	67.37	17.58			
		(Z)-2-Heptene	006443-92-1	17.89	4.67			
		Cyclopropane, trimethylmethylene-	034462-28-7	10.72	2.8			
		Hexane, 2,5-dimethyl-	000592-13-2	19.18	5.01			
		Hexane, 2,4-dimethyl-	000589-43-5	30.86	8.05			
		Cyclohexane, methyl-	000108-87-2	90.07	22.06			
		Pentane, 2,3,4-trimethyl-	000565-75-3	23.94	5.04			
		Heptane, 2-methyl-	000592-27-8	77.51	16.32			
		Heptane, 3-methyl-	000589-81-1	76.37	16.08			
		Cyclohexene, 1-methyl-	000591-49-1	13.35	3.48			
		Cyclohexane, 1,4-dimethyl-, cis-	000624-29-3	19.04	4.97			
		Toluene	000108-88-3	503.57	131.4	0.3983		
		Heptane, 2,6-dimethyl-	001072-05-5	12.49	3.26			
		Heptane, 2,5-dimethyl-	002216-30-0	22.74	5.93			
		Cyclopentane, propyl-	002040-96-2	11.88	3.1			
		Cyclohexane, ethyl-	001678-91-7	77.41	20.21			
		Heptane, 2,4-dimethyl-	002213-23-2	52.02	13.58			
		Octane, 3-methyl-	002216-33-3	29.53	7.71			
		Ethylbenzene	000100-41-4	207.84	47.08	0.2769		
		Xylene, m+p		525.55	119.06	2.9039		
		Styrene	000100-42-5	10.57	2.44	0.0697		
		Benzene, (1-methylethyl)-	000098-82-8	42.78	11.17			
		Nonane, 3-methyl-	005911-04-6	8.81	2.3			
		Benzene, propyl-	000103-65-1	68.57	13.72	3.6105		
		Benzene, 1-ethyl-3-methyl	000620-14-4	157.03	31.42			

		Benzene, 1-ethyl-4-methyl	000622-96-8	58.88	11.78		
		1R-α-Pinene	007785-70-8	5.86	1.03	0.0575	
		Benzene, 1,3,5-trimethyl-	000108-67-8	67.07	13.42		
		Benzene, 1-ethyl-2-methyl	000611-14-3	68.37	13.68		
		Benzene, 1,2,4-trimethyl-	000095-63-6	224.71	44.96	0.3747	
		Benzene, (1-methylpropyl)-	000135-98-8	16.21	4.23		
		Benzene, 1-methyl-2-(1-methylethyl)-	000527-84-4	27.99	7.31		
		Benzene, 1,2,3-trimethyl-	000-526-73-8	63.27	12.66	0.0745	
		Benzene, 1-methyl-3-propyl-	001074-43-7	34.31	8.96		
		Benzene, 1,2-diethyl-	000135-01-3	6.65	1.73		
		Benzene, 1-methyl-4-propyl-	001074-55-1	12.86	3.36		
		Benzene, 1-ethyl-2,4-dimethyl-	000874-41-9	33.58	8.76		
		Nonanal	000124-19-6	2.46	0.42	1.2233	
		Benzene, 2-ethyl-1,4-dimethyl-	001758-88-9	9	2.35		
		Benzene, 1,2,4,5-tetramethyl-	000095-93-2	11.23	2.93		
		Benzene, 1,2,3,4-tetramethyl-	000488-23-3	14.59	3.81		
		Benzene, 1-ethenyl-3-ethyl-	007525-62-4	5.86	1.53		
		Benzene, 2-ethenyl-1,4-dimethyl-	002039-89-6	5.38	1.4		
13.11.2017	Neurehefeld	Methane, chloro-	000074-87-3	122.43	31.96		1.3
		Ethyl chloride	000075-00-3	64.64	16.87		
		Ethanol	000064-17-5	740.63	193.32	0.3718	
		Methane, dimethoxy-	000109-87-5	100.15	26.14		
		Acetic acid, methyl ester	000079-20-9	219.84	57.38	0.0574	
		Ethane, 1,1-dimethoxy-	000534-15-6	33.16	8.66		
		Ethyl acetate	000141-78-6	138.4	37.78	0.0434	

		Methane, diethoxy-	000462-95-3	52.24	13.64		
		Heptane	000148-82-5	10.33	2.48	0.0037	
		Methyl isobutyl ketone	000108-10-1	59.14	14.2	0.0835	
		2-Butenoic acid, methyl ester, (E)-	000623-43-8	1.35	0.35		
		Toluene	000108-88-3	3.98	1.04	0.0032	
		Tetrachloroethylene	000127-18-4	2.63	0.69	0.0009	
		Xylene, m+p		4.33	0.98	0.0239	
		Cyclohexene, 4-ethenyl-1,4-dimethyl-	001743-61-9	3.43	0.9		
		Butane, 1,1,3,4-tetrachloro-1,2,2,3,4,4-hexafluoro-	000423-38-1	114.43	14.69		
		1-Propene, 3-chloro-1,1,2,3,3-pentafluoro-	000079-47-0	2.87	0.37		
		Butane,1,2,4-trichloro-heptafluoro-	000335-45-5	24.66	3.16		
		Decanal	000112-31-2	1.84	0.28	0.7079	
		Ethane, 1,1,2-trichloro-1,2,2-trifluoro-	000076-13-1	249.02	31.96		
27.12.2017	Neurehefeld	Ethanol	000064-17-5	5.34	1.39	0.0027	0.02
		Toluene	000108-88-3	2.76	0.72	0.0022	
		Xylene, m+p		2.65	0.6	0.0146	
6.2.2018	Neurehefeld	Ethyl acetate	000141-78-6	1.58	0.43	0.0005	0.04
		Butane, 2,2,3,3-tetramethyl-	000594-82-1	80.78	21.09		
		Toluene	000108-88-3	2.38	0.62	0.0019	
		Tetrachloroethylene	000127-18-4	3.29	0.86	0.0011	
		Styrene	000100-42-5	6.06	1.4	0.04	
28.11.2017	Nová Ves v Horách	Ethyl acetate	000141-78-6	1.07	0.29	0	0.2
		Hexane, 3-methyl-	000589-34-4	3.46	0.83	0.001	
		Heptane	000148-82-5	2.42	0.58	0.0009	
		1,4-Dioxane	000123-91-1	0.42	0.11		
		Toluene	000108-88-3	2.68	0.7	0.0021	
		Tetrachloroethylene	000127-18-4	8.58	2.24	0.0029	
		Xylene, m+p		3.13	0.71	0.0173	
		1R-α-Pinene	007785-70-8	13.32	2.35	0.1306	

		β-Pinene	000127-91-3	4.69	1.22	0.0371	
		Dodecane	000112-40-3	5.31	0.75	0.0068	
		1-Butene, 4,4-dichloro-1,1,2,3,3,4-hexafluoro-	000357-24-4	0.25	0.06		
		Butane, 1,1,3,4-tetrachloro-1,2,2,3,4,4-hexafluoro-	000423-38-1	3.57	0.93		
		Butane, 1,2,3,4-tetrachloro-1,1,2,3,4,4-hexafluoro-	000375-45-1	5.06	1.32		
20.11.2018	Nová Ves v Horách	2-butanon	000078-93-3	3.83	1.28	0.0456	2.51
		1-Butanol	000071-36-3	2.73	0.88	0.0233	
		Toluene	000108-88-3	8.2	2.14	0.0065	
		Xylene, m+p		4.72	1.07	0.0261	
		Hexanal	000066-25-1	1.63	0.39	1.3978	
		Nonanal	000124-19-6	2.03	0.34	1.0095	
22.1.2017	Olbernhau	Pentane, 3-methyl-	000096-14-0	1.94	0.54	0.0001	1.24
		Hexane, 2-methyl-	000591-76-4	1.67	0.4	0.001	
		Hexane, 3-methyl-	000589-34-4	1.08	0.26	0.0003	
		Toluene	000108-88-3	1.4	0.37	0.0011	
		Benzene, 1-methyl-4-(1-methylethyl)-	000099-87-6	1.42	0.25		
		Limonene	000138-86-3	1.18	0.21	0.0208	
		Decanal	000112-31-2	2.98	0.46	1.1471	
		Undecanal	000112-44-7	0.2	0.05	0.0102	
		Dodecanal	000112-54-9	0.44	0.12	0.055	
12.12.2017	Olbernhau	Toluene	000108-88-3	2.91	0.76	0.0023	0
		Tetrachloroethylene	000127-18-4	1.43	0.37	0.0005	
9.1.2018	Olbernhau	Toluene	000108-88-3	2.15	0.56	0.0017	0
15.3.2018	Olbernhau	Pentane, 2-methyl-	000107-83-5	6.24	1.74	0.0002	1.92
		Acetic acid	000064-19-7	12.95	3.38	0.5635	
		Cyclohexane	000110-82-7	48.95	14.58	0.0058	
		Toluene	000108-88-3	2.84	0.74	0.0022	
		Nonanal	000124-19-6	2.06	0.35	1.0244	
		Decanal	000112-31-2	0.84	0.13	0.3232	
26.1.2001	Sayda	Toluene	000108-88-3	0.5	0.13	0.0004	0.78
		Undecane	001120-21-4	3.51	0.54	0.0006	

		Dodecane	000112-40-3	5.03	0.56	0.0051	
		Decanal	000112-31-2	2.52	0.31	0.7698	
27.1.2017	Sayda	Toluene	000108-88-3	1.05	0.27	0.0008	4.02
		Benzene, 1-methyl-4-(1-methylethyl)-	000099-87-6	0.22	0.06		
		D-Limonene	005989-27-5	1.15	0.2	0.0203	
		Acetophenone	000098-86-2	1.55	0.31	0.1551	
		Undecane	001120-21-4	3.57	0.55	0.0006	
		Nonanal	000124-19-6	3.1	0.52	1.542	
		Dodecane	000112-40-3	5.84	0.65	0.0059	
		Decanal	000112-31-2	5.95	0.92	2.2903	
11.1.2017	Seiffen	1,3-Butadiene, 2-methyl-	000078-79-5	3.36	0.49	0.0102	1.01
		1-Propanol	000071-23-8	11.72	4.69	0.0499	
		Pentane, 2-methyl-	000107-83-5	8.17	2.28	0.0003	
		Benzene	000071-43-2	2.06	0.63	0.0002	
		Hexane, 3-methyl-	000589-34-4	5.17	1.24	0.0015	
		Toluene	000108-88-3	4.36	0.63	0.0019	
		Benzene, 1,3-dimethyl-	000108-38-3	0.53	0.08	0.0019	
		Decanal	000112-31-2	2.46	0.38	0.9469	
1.2.2017	Seiffen	Toluene	000108-88-3	0.46	0.12	0.0004	0
17.2.2017	Seiffen	2-Propenenitrile	000107-13-1	9.72	2.537		42.88
		Cyclopentane, methyl-	000096-37-7	4.06	1.16		
		Toluene	000108-88-3	7.26	1.895	0.0057	
		Hexanal	000066-25-1	1.06	0.255	9.091	
		Tetrachloroethylene	000127-18-4	4.72	0.685	0.0009	
		Heptane, 2,4-dimethyl-	002213-23-2	5.81	1.517		
		Hexane, 3-ethyl-	000619-99-8	2.46	0.641		
		Xylene, m+p		0.96	0.217	0.0043	
		Styrene	000100-42-5	3.92	0.905	0.0259	
		Benzaldehyde	000100-52-7	4.3	0.975	5.4139	
		Octanal	000124-13-0	1.12	0.21	21.009	
		D-Limonene	005989-27-5	1.42	0.251	0.0066	
		Undecane, 5,7-dimethyl-	017312-83-3	4.16	1.087		
		Undecane	001120-21-4	2.02	0.526	0.0006	
		Benzoic acid	000065-85-0	7.46	1.947	4.7478	
		Decanal	000112-31-2	5.1	0.785	1.9631	

		Naphthalene	000091-20-3	0.36	0.068	0.6141	
21.2.2017	Seiffen	2-Propenenitrile	000107-13-1	4.71	1.23	0.0002	0.85
		Pentane, 3-methyl-	000096-14-0	2.44	0.68	0.0001	
		Cyclopentane, methyl-	000096-37-7	2.59	0.74	0.0004	
		Toluene	000108-88-3	5.14	1.34	0.0041	
		Tetrachloroethylene	000127-18-4	3.6	0.52	0.0007	
		Hexane, 3-ethyl-	000619-99-8	1.7	0.44		
		Styrene	000100-42-5	1.34	0.31	0.0088	
		Undecane, 5,7-dimethyl-	017312-83-3	2.47	0.64		
		Decanal	000112-31-2	2.16	0.33	0.8314	
13.3.2017	Seiffen	2-Propenenitrile	000107-13-1	4.58	1.2		33.4
		Pentane, 2-methyl-	000107-83-5	2.72	0.76	0.0001	
		Pentane, 3-methyl-	000096-14-0	3.8	1.06	0.0001	
		Cyclopentane, methyl-	000096-37-7	2.38	0.68	0.0004	
		Hexane, 2-methyl-	000591-76-4	3.58	0.86	0.002	
		Toluene	000108-88-3	4.2	1.1	0.0033	
		Hexanal	000066-25-1	1.34	0.32	1.1492	
		Heptane, 2,4-dimethyl-	002213-23-2	5.44	1.42		
		Octane, 4-methyl-	002216-34-4	1.64	0.43		
		Styrene	000100-42-5	4.96	1.15	0.0327	
		α-Pinene	000080-56-8	1.08	0.19	0.8666	
		Benzaldehyde	000100-52-7	5.92	1.34	7.4536	
		Octanal	000124-13-0	1.08	0.2	20.2586	
		Undecane, 4,7-dimethyl-	017301-32-5	3.21	0.84		
		Acetophenone	000098-86-2	4.16	0.83	0.4163	
		Benzoic acid	000065-85-0	2.23	0.58	1.4187	
		Decanal	000112-31-2	4.66	0.72	1.7937	
27.3.2017	Seiffen	2-Propenenitrile	000107-13-1	16.65	4.35		62.5
		Pentane, 2-methyl-	000107-83-5	20.5	5.72	0.0008	
		Pentane, 3-methyl-	000096-14-0	23.51	6.56	0.0007	
		Cyclopentane, methyl-	000096-37-7	23.87	6.82	0.004	
		Hexane, 2-methyl-	000591-76-4	14	3.36	0.008	
		Benzene	000071-43-2	2.98	0.92	0.0003	
		Hexane, 3-methyl-	000589-34-4	22.25	5.34	0.0064	
		Heptane, 2-methyl-	000592-27-8	3.8	0.8	0.0073	
		Toluene	000108-88-3	14.48	3.78	0.0115	

		Octane	000111-65-9	7.31	1.54	0.0009	
		Hexanal	000066-25-1	2.78	0.67	2.3842	
		Acetic acid, butyl ester	000123-86-4	5.5	1.44	0.0897	
		Tetrachloroethylene	000127-18-4	23.5	5.32	0.0069	
		Heptane, 2,4-dimethyl-	002213-23-2	27.07	7.06		
		2,4-Dimethyl-1-heptene	019549-87-2	2.34	0.61		
		Heptane, 2,3-dimethyl-	003074-71-3	1.61	0.42		
		Octane, 4-methyl-	002216-34-4	14.87	3.88		
		Ethylbenzene	000100-41-4	1.74	0.39	0.0023	
		Xylene, m+p		2.5	0.57	0.0138	
		Styrene	000100-42-5	14.42	3.33	0.0951	
		Nonane	000111-84-2	4.9	0.92	0.0004	
		Heptanal	000111-71-7	2.24	0.47	2.6212	
		α-Pinene	000080-56-8	1.42	0.25	0.0139	
		Nonane, 2-methyl-	000871-83-0	5.84	1.52		
		Benzene, 1,3,5-trimethyl-	000108-67-8	2.6	0.52	0.0031	
		Benzaldehyde	000100-52-7	6.5	1.47	8.1839	
		Decane	000124-18-5	5.09	0.86	0.0014	
		Octanal	000124-13-0	2.04	0.38	38.2663	
		Benzene, 1,2,4-trimethyl-	000095-63-6	4.4	0.88	0.0073	
		Decane, 4-methyl-	002847-72-5	6.86	1.79		
		Limonene	000138-86-3	3.54	0.62	0.0164	
		Acetophenone	000098-86-2	4.68	0.94	0.4684	
		Undecane	001120-21-4	14.04	2.16	0.0025	
		Benzoic acid	000065-85-0	4.33	1.13	2.75	
		Dodecane	000112-40-3	11.19	1.58	0.0144	
		Decanal	000112-31-2	17.22	2.65	6.6284	
		Naphthalene	000091-20-3	0.52	0.1	0.887	
20.10.2017	Seiffen	Heptane	000148-82-5	1.33	0.32	0.0005	0.63
		1-Butanol	000071-36-3	2.26	0.73	0.0193	
		Toluene	000108-88-3	0.54	0.14	0.0004	
		Crotonic acid	003724-65-0	0.12	0.03		
		Styrene	000100-42-5	19.23	4.44	0.1269	
		Nonanal	000124-19-6	0.98	0.17	0.4873	
20.10.2017	Seiffen	Acetic acid, methyl ester	000079-20-9	48.28	12.6	0.0126	1.16
		1-Propanol	000071-23-8	0.9	0.23	0.0025	

one hour later		Ethane, 1,1-dimethoxy-	000534-15-6	21.79	5.69		
		Ethyl acetate	000141-78-6	221.04	60.33	0.0693	
		Hexane, 2-methyl-	000591-76-4	4.42	1.06	0.0025	
		Methane, diethoxy-	000462-95-3	41.64	10.87		
		Heptane	000148-82-5	45.42	10.9	0.0163	
		Methyl isobutyl ketone	000108-10-1	393.78	94.55	0.5562	
		Toluene	000108-88-3	1.99	0.52	0.0016	
		3-Penten-2-one, 4-methyl-	000141-79-7	1.02	0.27		
		Decane	000124-18-5	2.13	0.4	0.0006	
		Ethane, 1,2-dichloro-1,1,2,2-tetrafluoro-	000076-14-2	69.69	8.94		
		Butane, 1,2,3,4-tetrachloro-1,1,2,3,4,4-hexafluoro-	000375-45-1	200.9	25.79		
		Decanoic acid, nonadecafluoro-	000335-76-2	1.38	0.36		
		Butane,1,2,4-trichloro-heptafluoro-	000335-45-5	149.28	19.16		
		Butane, 1,1,3,4-tetrachloro-1,2,2,3,4,4-hexafluoro-	000423-38-1	1975.1	253.51		
		Ethane, 1,1,2,2-tetrachloro-1-fluoro-	000354-14-3	1118.51	143.56		
		Butane, 1,1,2,3,4,4-hexachloro-1,2,3,4-tetrafluoro-	000375-43-9	19.91	2.56		
		Decanal	000112-31-2	1.3	0.2	0.5002	
		Benzaldehyde, 3,4-dimethyl-	005973-71-7	2.53	0.66		
		Ethane, 1,1,2-trichloro-1,2,2-trifluoro-	000076-13-1	1054.84	135.39		
8.11.2017	Seiffen	Ethyl acetate	000141-78-6	254.28	69.41	0.0798	2.14
		Acetic acid	000064-19-7	35.86	9.36	1.5598	
		Methane, diethoxy-	000462-95-3	45.96	12		
		Benzene	000071-43-2	11.36	2.96	0.0011	
		Heptane	000142-82-5	37.83	9.08	0.0136	

		Methyl isobutyl ketone	000108-10-1	194.6	46.73	0.2749	
		Toluene	000108-88-3	7.51	1.96	0.0059	
		Xylene, m+p		5.47	1.24	0.0302	
		Styrene	000100-42-5	25.89	5.98	0.1709	
		Ethane, 1-chloro-1,1,2,2-tetrafluoro-	000354-25-6	24.53	3.15		
		Butane, 1,1,3,4-tetrachloro-1,2,2,3,4,4-hexafluoro-	000423-38-1	1002.59	128.68		
		Ethane, 1,1,2-trichloro-1,2,2-trifluoro-	000076-13-1	509.95	65.45		
		Butane,1,2,4-trichloro-heptafluoro-	000335-45-5	59.54	7.64		
		Butane, 1,2,3,4-tetrachloro-1,1,2,3,4,4-hexafluoro-	000375-45-1	636.86	81.74		
28.11.2017	Seiffen	Methane, chloro-	000074-87-3	237.47	61.98		11.61
		Ethane, methoxy-	000540-67-0	56.48	14.74		
		Ethyl chloride	000075-00-3	125.08	32.65		
		Ethyl ether	000060-29-7	34.51	9.01		
		Methane, dimethoxy-	000109-87-5	67.56	17.64		
		Acetic acid, methyl ester	000079-20-9	242.21	63.22	0.06	
		Ethyl acetate	000141-78-6	227.82	62.18	0.07	
		Hexane, 3-methyl-	000589-34-4	23.58	5.66	0.01	
		Methane, diethoxy-	000462-95-3	101.61	26.52		
		Heptane	000148-82-5	22.58	5.42	0.01	
		1-Butanol	000071-36-3	7.24	2.35	0.06	
		2-Propanol, 1-methoxy-	000107-98-2	13.42	3.5		
		Pentanal	000110-62-3	2.12	0.59	1.44	
		Cyclohexane, methyl-	000108-87-2	2.37	0.58	0	
		Methyl isobutyl ketone	000108-10-1	122.48	29.41	0.17	
		2-Butenoic acid, methyl ester, (E)-	000623-43-8	5.11	1.33		
		Toluene	000108-88-3	5.13	1.34	0	
		Acetic acid, butyl ester	000123-86-4	155.56	32.21	2.01	
		Hexanal	000066-25-1	5.8	1.39	4.97	

		2-Butenoic acid, ethyl ester	010544-63-5	7.5	1.96		
		Ethylbenzene		3.53	0.8	0	
		Xylene, m+p		13.24	3	0.07	
		2-Propanol, 1-ethoxy-	001569-02-4	2.31	0.6		
		1R-α-Pinene	007785-70-8	2.78	0.49	0.03	
		2-Cyclopenten-1-one, 2-methyl-	001120-73-6	1.86	0.49		
		Benzene, 1,2,4-trimethyl-	000095-63-6	5.2	1.04	0.01	
		Undecane	001120-21-4	12.22	1.88	0	
		Nonanal	000124-19-6	3.8	0.64	1.89	
		Decanal	000112-31-2	2.02	0.31	0.78	
		Butane, 1,1,3,4-tetrachloro-1,2,2,3,4,4-hexafluoro-	000423-38-1	474.52	60.91		
		Butane, 1,2,3,4-tetrachloro-1,1,2,3,4,4-hexafluoro-	000375-45-1	101.54	13.03		
		Ethane, 1,2-dichloro-1,1,2,2-tetrafluoro-	000076-14-2	20.36	2.61		
		Butane,1,2,4-trichloro-heptafluoro-	000335-45-5	40.66	5.22		
		1-Butene, 4,4-dichloro-1,1,2,3,3,4-hexafluoro-	000357-24-4	22.63	2.9		
		1-Propene, 3-chloro-1,1,2,3,3-pentafluoro-	000079-47-0	192.98	24.77		
		Ethane, 1,1,2-trichloro-1,2,2-trifluoro-	000076-13-1	936.4	120.19		
25.1.2018	Seiffen	Ethyl chloride	000075-00-3	1.46	0.38		3
		Ethanol	000064-17-5	68.24	17.81	0.0343	
		Acetic acid, methyl ester	000079-20-9	14.3	3.73	0.0037	
		Ethane, 1,1-dimethoxy-	000534-15-6	0.35	0.09		
		Ethyl acetate	000141-78-6	53.16	14.51	0.0167	
		Ethane, 1-ethoxy-1-methoxy-	010471-14-4	2.28	0.59		
		Methane, diethoxy-	000462-95-3	1.05	0.27		
		Methyl isobutyl ketone	000108-10-1	3.38	0.81	0.0048	

		Toluene	000108-88-3	1.92	0.5	0.0015	
		Propanoic acid, 2-methyl-	000079-31-2	0.85	0.22	0.1473	
		Acetic acid, butyl ester	000123-86-4	209.9	43.46	2.7161	
		Ethylbenzene	000100-41-4	3.53	0.8	0.0047	
		Xylene, m+p		13.51	3.06	0.0746	
		Butane,1,2,4-trichloro-heptafluoro-	000335-45-5	367.01	47.11		
		Butane, 1,2,3,4-tetrachloro-1,1,2,3,4,4-hexafluoro-	000375-45-1	60.76	7.8		
		Ethane, 1,2-dichloro-1,1,2,2-tetrafluoro-	000076-14-2	3.95	0.51		
		Ethane, 1,1,2-trichloro-1,2,2-trifluoro-	000076-13-1	618.48	79.38		
		Butane, 1,1,3,4-tetrachloro-1,2,2,3,4,4-hexafluoro-	000423-38-1	197.52	25.35		
26.1.2018	Seiffen	Ethane, methoxy-	000540-67-0	4.45	1.16		0.11
		Ethyl chloride	000075-00-3	17.76	4.64		
		Ethanol	000064-17-5	73.99	19.31	0.0371	
		Ethane, 1,2-diethoxy-	000629-14-1	4.8	1.25		
		Methane, dimethoxy-	000109-87-5	25.92	6.76		
		Acetic acid, methyl ester	000079-20-9	51.96	13.56	0.0136	
		Hexane	000110-54-3	6.12	1.6	0.0011	
		Ethyl Acetate	000141-78-6	47.32	12.92	0.0148	
		Methane, diethoxy-	000462-95-3	6.18	1.61		
		Heptane	000148-82-5	2.92	0.7	0.001	
		Methyl isobutyl ketone	000108-10-1	15.42	3.7	0.0218	
		Toluene	000108-88-3	2.07	0.54	0.0016	
		Acetic acid, butyl ester	000123-86-4	1.2	0.25	0.0155	
		Butane, 1,1,3,4-tetrachloro-1,2,2,3,4,4-hexafluoro-	000423-38-1	62.88	8.07		
		1-Propene, 3-chloro-1,1,2,3,3-pentafluoro-	000079-47-0	4.48	0.58		

		Butane,1,2,4-trichloro-heptafluoro-	000335-45-5	10.16	1.3		
		Butane, 1,2,3,4-tetrachloro-1,1,2,3,4,4-hexafluoro-	000375-45-1	6.28	0.81		
		Ethane, 1,2-dichloro-1,1,2,2-tetrafluoro-	000076-14-2	0.35	0.05		
		1-Butene, 4,4-dichloro-1,1,2,3,3,4-hexafluoro-	000357-24-4	4.55	0.58		
		Ethane, 1,1,2-trichloro-1,2,2-trifluoro-	000076-13-1	120.88	15.52		
18.2.2018	Seiffen	Methane, chloro-	000074-87-3	18.04	4.71		0.12
		Ethane, methoxy-	000540-67-0	4.21	1.1		
		Ethyl chloride	000075-00-3	21.54	5.62		
		Ethanol	000064-17-5	56.41	14.72	0.0283	
		Ethyl ether	000060-29-7	5.12	1.34		
		Acetic acid, methyl ester	000079-20-9	66.67	17.4	0.0174	
		Methylene chloride	000075-09-2	3.31	0.86	0	
		Ethyl acetate	000141-78-6	70.04	19.12	0.022	
		Ethane, 1-ethoxy-1-methoxy-	010471-14-4	20.99	5.48		
		Methane, diethoxy-	000462-95-3	10	2.61		
		Methyl isobutyl ketone	000108-10-1	29.26	7.03	0.0413	
		Toluene	000108-88-3	3.68	0.96	0.0029	
		Xylene, m+p		2.21	0.5	0.0122	
		Ethane, 1,2-dichloro-1,1,2,2-tetrafluoro-	000076-14-2	2.53	0.32		
		Ethane, 1,1-dichloro-1,2,2,2-tetrafluoro-	000374-07-2	1.9	0.24		
		1-Butene, 4,4-dichloro-1,1,2,3,3,4-hexafluoro-	000357-24-4	5.18	0.66		
		1-Propene, 3-chloro-1,1,2,3,3-pentafluoro-	000079-47-0	1.16	0.15		
		Butane, 1,1,3,4-tetrachloro-1,2,2,3,4,4-hexafluoro-	000423-38-1	117.85	15.13		

		Butane,1,2,4-trichloro-heptafluoro-	000335-45-5	19.87	2.55		
		Butane, 1,2,3,4-tetrachloro-1,1,2,3,4,4-hexafluoro-	000375-45-1	11.16	1.43		
		Butane, 1,1,2,3,4,4-hexachloro-1,2,3,4-tetrafluoro-	000375-43-9	4.53	0.58		
		Ethane, 1,1,2-trichloro-1,2,2-trifluoro-	000076-13-1	136.96	17.58		
5.3.2018	Seiffen	Methane, chloro-	000074-87-3	18.04	4.71		0.12
		Ethane, methoxy-	000540-67-0	4.21	1.1		
		Ethyl chloride	000075-00-3	21.54	5.62		
		Ethanol	000064-17-5	56.41	14.72	0.0283	
		Ethyl ether	000060-29-7	5.12	1.34		
		Acetic acid, methyl ester	000079-20-9	66.67	17.4	0.0174	
		Methylene chloride	000075-09-2	3.31	0.86	0.0000	
		Ethyl acetate	000141-78-6	70.04	19.12	0.0220	
		Ethane, 1-ethoxy-1-methoxy-	010471-14-4	20.99	5.48		
		Methane, diethoxy-	000462-95-3	10	2.61		
		Methyl isobutyl ketone	000108-10-1	29.26	7.03	0.0413	
		Toluene	000108-88-3	3.68	0.96	0.0029	
		Xylene, m+p		2.21	0.5	0.0122	
		Ethane, 1,2-dichloro-1,1,2,2-tetrafluoro-	000076-14-2	2.53	0.32		
		Ethane, 1,1-dichloro-1,2,2,2-tetrafluoro-*	000374-07-2	1.9	0.24		
		1-Butene, 4,4-dichloro-1,1,2,3,3,4-hexafluoro-*	000357-24-4	5.18	0.66		
		1-Propene, 3-chloro-1,1,2,3,3-pentafluoro-	000079-47-0	1.16	0.15		
		Butane, 1,1,3,4-tetrachloro-1,2,2,3,4,4-hexafluoro-	000423-38-1	117.85	15.13		
		Butane,1,2,4-trichloro-heptafluoro-	000335-45-5	19.87	2.55		

		Butane, 1,2,3,4-tetrachloro-1,1,2,3,4,4-hexafluoro-	000375-45-1	11.16	1.43		
		Butane, 1,1,2,3,4,4-hexachloro-1,2,3,4-tetrafluoro-	000375-43-9	4.53	0.58		
		Ethane, 1,1,2-trichloro-1,2,2-trifluoro-	000076-13-1	136.96	17.58		
6.11.2018	Seiffen	Ethanol	000064-17-5	101.58	26.51	0.051	3.03
		Ethane, 1,1-dimethoxy-	000534-15-6	0.86	0.22		
		Ethyl acetate	000141-78-6	5.58	1.52	0.0018	
		Ethane, 1-ethoxy-1-methoxy-	010471-14-4	7.91	2.06		
		Acetic acid, methylethyl ester	000108-21-4	19.18	5.01	0.0313	
		Methane, diethoxy-	000462-95-3	1.36	0.35		
		1-Butanol	000071-36-3	2.46	0.8	0.021	
		Methyl isobutyl ketone	000108-10-1	9.6	2.31	0.0136	
		Toluene	000108-88-3	4.44	1.16	0.0035	
		Acetic acid, butyl ester	000123-86-4	124	25.67	1.6046	
		Butanoic acid	000107-92-6	0.88	0.23	1.2114	
		Nonane	000111-84-2	3.84	0.72	0	
		Xylene, m+p		4.86	1.1	0.0268	
		Styrene	000100-42-5	9.09	2.1	0.06	
		Limonene	000138-86-3	1.24	0.22	0.0058	
		Ethane, 1,1,2-trichloro-1,2,2-trifluoro-	000076-13-1	10.98	1.41		
12.11.2018	Seiffen	Ethyl acetate	000141-78-6	254.28	69.41	0.0798	2.14
		Acetic acid	000064-19-7	35.86	9.36	1.5598	
		Ethane, 1-ethoxy-1-methoxy-	010471-14-4	36.29	9.47		
		Methane, diethoxy-	000462-95-3	45.96	12		
		Benzene	000071-43-2	11.36	2.96	0.0011	
		Heptane	000142-82-5	37.83	9.08	0.0136	
		Methyl isobutyl ketone	000108-10-1	194.6	46.73	0.2749	
		Toluene	000108-88-3	7.51	1.96	0.0059	
		Xylene, m+p		5.47	1.24	0.0302	

		Styrene	000100-42-5	25.89	5.98	0.1709	
		Ethane, 1-chloro-1,1,2,2-tetrafluoro-	000354-25-6	24.53	3.15		
		Butane, 1,1,3,4-tetrachloro-1,2,2,3,4,4-hexafluoro-	000423-38-1	1002.59	128.68		
		Butane, 1,2,3,4-tetrachloro-1,1,2,3,4,4-hexafluoro-	000375-45-1	509.95	65.45		
		Butane,1,2,4-trichloro-heptafluoro-	000335-45-5	59.54	7.64		
		Ethane, 1,1,2-trichloro-1,2,2-trifluoro-	000076-13-1	636.86	81.74		
28.11.2018	Seiffen	Ethyl acetate	000141-78-6	41.46	11.32	0.01	3.46
		2-Butanone	000078-93-3	53.46	17.83	0.64	
		1-Butanol	000071-36-3	1.4	0.45	0.01	
		Toluene	000108-88-3	12.64	3.3	0.01	
		Acetic acid, butyl ester	000123-86-4	190.3	39.4	2.46	
		Octane, 4-methyl-	002216-34-4	2.64	0.69		
		Ethylbenzene	000100-41-4	6.45	1.46	0.01	
		Xylene, m+p		22.07	5	0.12	
		Styrene	000100-42-5	7.79	1.8	0.05	
		Benzene,1-ethyl-3-methyl-	143314-17-4	9.42	1.9	0.11	
		Benzene, 1,2,4-trimethyl-	000095-63-6	20.69	4.14	0.03	
14.2.2017	Vřesová	Pentane, 2-methyl-	000107-83-5	2.47	0.69	0.0001	1.11
		Pentane, 3-methyl-	000096-14-0	9.07	2.53	0.0003	
		Acetic acid	000064-19-7	3.06	0.8	0.1332	
		1,4-Dioxane	000123-91-1	0.61	0.16		
		Toluene	000108-88-3	2.91	0.76	0.0023	
		Tetrachloroethylene	000127-18-4	2.26	0.33	0.0004	
		Heptane, 2,4-dimethyl-	002213-23-2	1.44	0.38		
		Octane, 4-methyl-	002216-34-4	0.64	0.17		
		Undecane, 4,7-dimethyl-	017301-32-5	1.21	0.32		
		Decanal	000112-31-2	2.54	0.39	0.9777	
		Nonadecane	000629-92-5	0.67	0.17		
30.3.2017	Vřesová	Toluene	000108-88-3	2.13	0.56	0.0017	1.78

		Nonanal	000124-19-6	2.01	0.34	0.9996	
		Decanal	000112-31-2	2.03	0.31	0.7814	

Table S4. Hazard Index (HI) for chronic non-carcinogenic effects from exposure to chemicals during the odor episodes near the Czech-German border.

**DATE**	**SITE**	**HC**	**C_3-C_4**	**HAL HC**	**HCO**	**OH**	**ACIDS**	**ESTERS**	**TERP**	**2-PRCN**
10.11.2017	Háj u Duchcova	0.011		0.001		0.001	0.049			
16.11.2017	Háj u Duchcova	0.007				0.001	0.908			
24.11.2017	Háj u Duchcova	0.041		0.001						
26.11.2017	Háj u Duchcova	0.227	0.000	0.000		0.001		0.003		
3.12.2017	Háj u Duchcova	0.001								
1.1.2018	Háj u Duchcova	0.006		0.003					0.002	
11.1.2018	Háj u Duchcova	0.005		0.001						
7.3.2018	Háj u Duchcova	0.007	0.001	0.001						
30.10.2018	Háj u Duchcova	0.015	0.000	0.007					0.000	
7.11.2018	Háj u Duchcova	0.004				0.001				
16.11.2018	Háj u Duchcova	0.007		0.001		0.001				
8.2.2017	Kühnheide	0.008		0.009				0.000	0.000	
4.2.2017	Litvínov	0.001				0.003				
16.11.2018	Litvínov	0.005	0.001	0.000		0.007				
20.11.2018	Litvínov	0.007	0.001			0.001				
8.2.2017	Marienberg	0.016		0.006					0.001	
23.1.2018	Neuhausen	0.081							0.001	
10.3.2018	Neuhausen	0.003	0.048	0.003		0.043			0.006	

24.3.2018	Neuhausen	3.960				0.071			0.003	
13.11.2017	Neurehefeld	0.016	0.047	1.142		0.190		0.415		
27.12.2017	Neurehefeld	0.005				0.001				
6.2.2018	Neurehefeld	0.030		0.012				0.003		
28.11.2017	Nová Ves v Horách	0.011	0.001	0.023				0.002	0.007	
22.1.2017	Olbernhau	0.004							0.001	
8.11.2017	Olbernhau	0.180				0.006		0.035	0.002	
28.11.2017	Olbernhau	0.061	0.089	0.470	0.000	0.005		0.557	0.001	
11.12.2017	Olbernhau	0.022	0.012			0.031		0.028		
11.12.2017	Olbernhau	0.002		0.005						
9.1.2018	Olbernhau	0.001								
15.3.2018	Olbernhau	0.015					0.075			
26.1.2017	Sayda	0.013								
27.1.2017	Sayda	0.014							0.001	
11.1.2017	Seiffen	0.017				0.003				
1.2.2017	Seiffen	0.000								
17.2.2017	Seiffen	0.044		0.017					0.001	0.704
21.2.2017	Seiffen	0.010		0.013						0.341
13.3.2017	Seiffen	0.027							0.001	0.332
27.3.2017	Seiffen	0.177		0.085	1.081			0.001	0.001	1.205
20.10.2017	Seiffen	0.028				0.001				
20.10.2017	Seiffen	0.016	0.158	0.022		0.051		0.469		
25.1.2018	Seiffen	0.022	0.002	0.033		0.010	0.123	0.145		
26.1.2018	Seiffen	0.003	0.007	0.259		0.011		0.013		
18.2.2018	Seiffen	0.001						0.098		
5.3.2018	Seiffen	0.017	0.016	0.344		0.008		0.162		
6.11.2018	Seiffen	0.028	0.004	0.000		0.016	0.208	0.047	0.001	
12.11.2018	Seiffen	0.112	0.082	0.012				0.526		
28.11.2018	Seiffen	0.100	0.013			0.001		0.115		
14.2.2017	Vřesová	0.008	0.001	0.006						
30.3.2017	Vřesová	0.001								

Abbreviations: **HC**= Complex mixture of aliphatic and aromatic hydrocarbons; **C_3-C_4**= C_3-C_4 hydrocarbons, ketones, esters; **PETROL**= petroleum hydrocarbons; **HAL HC**= halogenated hydrocarbons; **HCO**= aldehydes; **OH**= alcohols; **TERP**= terpenes; **2-PRCN**= 2-propenenitrile.
